# Identification of gene fusion events in *Mycobacterium tuberculosis* that encode chimeric proteins

**DOI:** 10.1093/nargab/lqaa033

**Published:** 2020-05-18

**Authors:** James Gallant, Jomien Mouton, Roy Ummels, Corinne ten Hagen-Jongman, Nastassja Kriel, Arnab Pain, Robin M Warren, Wilbert Bitter, Tiaan Heunis, Samantha L Sampson

**Affiliations:** 1 DST/NRF Centre of Excellence for Biomedical Tuberculosis Research, South African Medical Research Council Centre for Tuberculosis Research, Division of Molecular Biology and Human Genetics, Department of Biomedical Science, Faculty of Medicine and Health Science, Stellenbosch University, Tygerberg, Cape Town 7505, South Africa; 2 Section of Molecular Microbiology, Amsterdam Institute for Molecules, Medicines and Systems, Vrije Universiteit Amsterdam, 1081 HZ Amsterdam, The Netherlands; 3 Medical Microbiology and Infection Control, Vrije Universiteit Amsterdam, Amsterdam UMC, 1081 HZ Amsterdam, The Netherlands; 4 Biological and Environmental Sciences and Engineering (BESE) Division, King Abdullah University of Science and Technology, Thuwal 23955-6900, Kingdom of Saudi Arabia; 5 Global Station for Zoonosis Control, GI-CoRE, Hokkaido University, 001-0020, N20 W10 Kita-ku, Sapporo, Japan; 6 Biosciences Institute, Faculty of Medical Sciences, Newcastle University, Newcastle upon Tyne NE2 4HH, UK

## Abstract

*Mycobacterium tuberculosis* is a facultative intracellular pathogen responsible for causing tuberculosis. The harsh environment in which *M. tuberculosis* survives requires this pathogen to continuously adapt in order to maintain an evolutionary advantage. However, the apparent absence of horizontal gene transfer in *M. tuberculosis* imposes restrictions in the ways by which evolution can occur. Large-scale changes in the genome can be introduced through genome reduction, recombination events and structural variation. Here, we identify a functional chimeric protein in the *ppe38–71* locus, the absence of which is known to have an impact on protein secretion and virulence. To examine whether this approach was used more often by this pathogen, we further develop software that detects potential gene fusion events from multigene deletions using whole genome sequencing data. With this software we could identify a number of other putative gene fusion events within the genomes of *M. tuberculosis* isolates. We were able to demonstrate the expression of one of these gene fusions at the protein level using mass spectrometry. Therefore, gene fusions may provide an additional means of evolution for *M. tuberculosis* in its natural environment whereby novel chimeric proteins and functions can arise.

## INTRODUCTION

*Mycobacterium tuberculosis*, the causative agent of the disease tuberculosis, is a facultative intracellular pathogen. Adaptation to an intracellular niche is typically accompanied by reductive evolution, which favours the accumulation of pseudogenes and gene deletions. These events are prevalent in bacterial pathogens (1) and mutualistic endosymbiotic bacteria (2) and are observed in obligate pathogenic mycobacteria (3–5). Reductive evolution is most commonly associated with the loss of redundant genetic material, as a pathogen or symbiont transitions towards an intracellular lifestyle (4). As an intracellular species, *M. tuberculosis* experiences a constant selective pressure in competition with the host, even though genome reduction in this pathogen has been limited. In order to maintain a competitive advantage, evolutionary changes are required. Usually, lateral evolution (horizontal gene transfer) plays an important role in bacterial adaptation to specific environments (6). Horizontal gene transfer is crucial to asexually reproductive organisms as this process provides a mechanism for the incorporation of genetic diversity in response to a dynamic environment (7,8). This phenomenon likely occurs due to isolation of the bacilli while residing within a specialized environment such as a human macrophage. It has previously been shown that horizontal gene transfer occurs at higher frequency between closely related organisms who share the same niche (9,10). However, horizontal gene transfer is limited or even absent in intracellular pathogens, likely due to isolation of the bacilli while residing within a specialized intracellular environment. In line with this, *M. tuberculosis* displays minimal signs of recent horizontal gene transfer events in the genome (6). *Mycobacterium tuberculosis* relies on mechanisms independent of lateral evolution to acquire new material that facilitates continued evolution, such as genome recombination, gene duplication events and single nucleotide variants (11). While the contribution of single nucleotide variants in *M. tuberculosis* evolution and adaptation has been well characterized (12), the functional contribution of large-scale genomic variation is largely understudied.

It was recently reported that a multi-operon deletion in the *ppe38–71* operon of *M. tuberculosis* had a major effect on the surface characteristics of this pathogen, as it resulted in the loss of all secreted proline–glutamic acid polymorphic GC-rich sequence (PE-PGRS) and PPE major polymorphic tandem repeat proteins (13). This was especially striking due to the deletion occurring naturally within members of the highly successful Beijing strain family of *M. tuberculosis* (13). Of interest was the specific nature of this deletion, where the breakpoints fell within the open reading frames of distally located genes. We thus hypothesized that this type of rearrangement can result in the formation of novel chimeric proteins. However, this process will only produce a functional chimeric protein if the frame is maintained when creating gene fusions. This phenomenon has garnered much attention in the cancer research field, where cells are prone to large-scale rearrangements in the genome (14–16), and similar mechanisms have recently been demonstrated in bacteria (17,18). We reasoned that the formation of natural chimeric proteins provides a mechanism for functional large-scale alterations in the genome in the absence of horizontal gene transfer.

We have previously used comparative genomics in conjunction with discovery-based proteomics to create custom proteome search databases for analysing strain-specific features in clinical *M. tuberculosis* isolates (19). Here, we expand on our approach and extend it to naturally occurring gene fusions by designing and implementing custom software to identify fusions in the genomes of *M. tuberculosis* clinical isolates. In addition, we were able to confirm the expression of fusion proteins using tandem mass spectrometry. We demonstrate an additional means for *M. tuberculosis* evolution, which is likely applicable to the adaptation of other intracellular pathogens as well.

## MATERIALS AND METHODS

### Bacterial culture

*Mycobacterium tuberculosis* CDC1551 and H37Rv were used as wild-type strains; *Δppe38–71* and *Δppe38–71::*pMVHSP60-*ppe38–71* (complemented strain) were generated previously from *M. tuberculosis* CDC1551 as the parental strain (13). Clinical isolates of *M. tuberculosis* used in this study were obtained from the South African Western Cape region. Procurement of clinical isolates was approved by the Stellenbosch University Health Research Ethics Committee (approval number N10/04/126). Samples were de-identified and not linked to any patient information. Samples obtained from patients were initially cultured in mycobacterial growth indicator tubes (MGITs) at 37°C until growth was detected by means of a BACTEC 960 broth culture system (BD Biosciences, NJ, USA). Positive MGIT cultures were centrifuged, stored as glycerol cryobead stocks in the Division of Molecular Biology and Human Genetics strain bank as bacterial seed lots or represented by genomic DNA.

For further experimentation, mycobacterial cultures were cultured in either modified Sauton’s media (0.4% l-asparagine, 0.4% glucose, 0.2% citric acid, 0.05% monopotassium phosphate, 0.05% magnesium sulfate, 0.005% ferric ammonium citrate, 0.1 ml of 1% zinc sulfate and 0.05% Tween 80, pH 7.0) or Middlebrook 7H9 media supplemented with an oleic acid, albumin, dextrose and catalase (OADC) mix (BD Biosciences, NJ, USA), 0.5% glycerol and 0.05% Tween 80. Mycobacterial cultures were grown without shaking at 37°C until harvesting. Long-term storage of mycobacterial cultures was done at −80°C in 20% glycerol.

*Escherichia coli* Top10F′ cells were cultured in lysogeny broth (LB) (1% Bacto-tryptone, 0.5% Bacto-yeast extract, 1% sodium chloride) media with shaking at 37°C, or kept at −80°C in 20% glycerol until use.

### Mycobacterial strain selection

Twenty-one *M. tuberculosis* strains were selected for initial analysis (Supplementary Table S1); 17 of these were genotyped based on spoligotyping and restriction fragment length polymorphism was used to stratify whether the lineage 2 strains were typical or atypical as previously described (20,21), while the remaining 4 strains (HN878, S1945, S2135 and S2701) were genotyped in previous studies (13,22). These specific strains were chosen based on their genetically diverse profile from two commonly occurring mycobacterial lineages in the South African Western Cape region and formed the basis of our study (Supplementary Figure S1A in Supplementary File S1). An additional 159 clinical isolates were chosen from an in-house mycobacterial genome sequence repository for a total of 180 *M. tuberculosis* clinical isolates (Supplementary Figure S1B in Supplementary File S1). In total, 90 independently isolated strains from each lineage were procured to gain greater statistical power during computational analysis. These samples have a genetic bottleneck due to the limited sites where *M. tuberculosis* isolates can be sampled. To account for this, the lineages were determined *in silico* using TBProfiler to avoid including multiple strains that represent a single sub-lineage (23).

### DNA extraction and whole genome sequencing

Genomic DNA was extracted as previously described (24). The DNA library was sequenced on an Illumina HiSeq2000 platform (Illumina, Inc., CA, USA). Briefly, 1 μg of DNA was used for library preparation using the Nextera DNA sample preparation kit according to the manufacturer’s instructions. Paired-end reads were sequenced using ∼500 bp fragment sizes. FastQ files for previously sequenced clinical isolates used in this study were obtained from the Stellenbosch University mycobacterial genome sequence repository.

### Construction of genomics pipeline

We have developed an in-house automated data analysis pipeline using the Bash scripting language on the Ubuntu distribution of Linux for the detection of large deletions and potential gene fusions. Illumina FastQ files were processed in accordance with the genome analysis toolkit (GATK) best practices guide using either *M. tuberculosis* H37Rv (NC_000962.3) or *M. tuberculosis* CDC1551 (AE000516.2) as the reference strain (25,26). FastQ files were trimmed and aligned using BWA and NovoAlign software (27). The resulting binary alignment map (BAM) files were used for single nucleotide variation detection using GATK and SAMtools (25,28). Automatic *in silico* lineage typing was determined by piping raw FastQ files to TBProfiler (23). Detection of deletions by read pair and split reads was done using Delly and Lumpy, while BEDTools was used to detect deletions based on zero coverage (29–31).

### Construction of gene fusion identification pipeline

To identify gene fusions, we first extracted a list of all the structural variants (SVs) found within the genome. All SVs used for high-throughput gene fusion detection were obtained from either Delly (29) or Lumpy (30). These include insertions, deletions, duplications, inversions and translocations. This list of SVs was further filtered for deletions, as these had the highest likelihood of recombining into gene fusions. The breakpoints of these deletions are subsequently annotated against the reference database with either the affected gene or the intergenic region. Using this information, we applied a set of filtering parameters to identify a list of putative gene fusions.

First, we excluded any deletions that did not span multiple genes. If this criterion was passed, the deletion breakpoints had to fall within open reading frames of genes on each side of the breakpoints, and these genes had to be in the same orientation. If all these criteria were met, 2000 bp flanking each side of the breakpoint was extracted from the BAM files and converted to FastQ format. This format was used to determine optimal *k*-mers using kmergenie and *de novo* assembled using SOAPdenovo2, which allows for precise breakpoint detection (32,33). The resulting contigs were ordered against the corresponding truncated reference sequence of *M. tuberculosis* H37Rv using ABACAS, which results in a consensus sequence (34). Post-pipeline analysis included manually inspecting the potential fusion sequence for open reading frames by searching in six frames using NCBI ORF finder and translated to protein sequence (35). This amino acid sequence from each potential fusion protein was cross-referenced to both parent amino acid sequences. If a match occurs, and no insertion sequences are present in the genomic region, the fusion amino acid sequences were added to a protein FASTA database. This database was ultimately used for downstream peptide identification by mass spectrometry.

### Harvesting of whole cell lysates and supernatants

*Mycobacterium tuberculosis* isolates were cultured in modified Sauton’s media supplemented with Tween 80 and allowed to propagate for 7 days at 37°C until an OD_600_ of 1.0 without shaking. *Mycobacterium tuberculosis Δppe38–71* and the complemented strain strains were supplemented with either hygromycin (50 μg/ml) or kanamycin (25 μg/ml) and hygromycin (50 μg/ml), respectively. Antibiotics were omitted during growth for all clinical isolates. Cultures were washed three times with phosphate-buffered saline (PBS) to remove residual Tween 80 and inoculated in modified Sauton’s media without Tween 80 at an OD_600_ of 0.05. Bacterial cultures were allowed to propagate for an additional 7 days. Not all clinical isolates defined in Supplementary Table S1 were able to grow in the modified Sauton’s media, likely due to the lack of OADC, and were therefore omitted when determining PE-PGRS secretion. The bacterial cells and supernatant were separated by centrifugation (4000 rpm, 10 min) followed by resuspension of the cell pellet in lysis buffer [8 M urea in 100 mM tetraethylammonium bromide (TEAB), 5 mM tris(2-carboxyethyl)phosphine (TCEP), Benzonase, Roche cOmplete™ EDTA-free cocktail tablets]. Cell-free supernatants were filter sterilized using a 0.22 μM Steriflip filter unit (Merck Millipore, MA, USA). Whole cell lysates were prepared by bead beating the resuspended cell pellet (20 s cycles with 20 s on ice for a total of eight cycles), followed by clarification (14 000 rpm, 10 min, 4°C). Sterilized cell-free supernatants were concentrated with Amicon ultra-15 kDa spin filters (Merck Millipore, MA, USA), to ∼200 μl by centrifugation (14 000 rpm, 4°C). Concentrated cell-free supernatants were precipitated overnight at −20°C using four volumes of ice-cold acetone. Protein content was quantified using a modified version of the Bradford protein assay (36).

### Sample preparation and liquid chromatography–tandem mass spectrometry

Whole cell lysates of *M. tuberculosis* clinical isolates S507, S5527 and S5218 were prepared in biological triplicate as mentioned earlier and processed for liquid chromatography–tandem mass spectrometry (LC–MS/MS). Whole cell lysates were digested to peptides for shotgun proteomics following an in-solution digestion protocol. Briefly, equal concentration of proteins resuspended in urea buffer (8 M urea in 100 mM TEAB) (Sigma-Aldrich, MO, USA) were reduced with 5 mM TCEP (Sigma-Aldrich, MO, USA) and alkylated with 5.5 mM iodoacetamide (Sigma-Aldrich, MO, USA) in the dark for 1 h, respectively. The protein solution was diluted with four volumes of 50 mM TEAB (Sigma-Aldrich, MO, USA) to contain a concentration of <2 M urea. Proteins were digested to peptides by addition of a 1:50 ratio of sequencing grade modified trypsin (Promega, WI, USA) to total protein content and incubated in a humidified chamber at 37°C for 18 h. The resulting peptide mix was dried using a desiccator and resuspended in 2% acetonitrile supplemented with 0.1% formic acid. Peptides were desalted using stop and go extraction tips as described previously (37). The desalted peptides were dried in a desiccator and resuspended in 2% acetonitrile supplemented with 0.1% formic acid before mass spectrometry analysis.

A total of 1 μg of peptides from each sample was analysed, independently, on an Orbitrap Fusion Tribid mass spectrometer (Thermo Fisher Scientific, MA, USA) connected to a Thermo Scientific UltiMate 3000 RSLCnano system (Thermo Fisher Scientific, MA, USA). Peptides were injected on a PepMap C18 LC pre-column (300 μm ID × 5 mm, 5 μm, 300 Å) followed by separation on an analytical column packed with C18 Aeris peptide 3.6 μM beads at a 500 nl/min flow rate. Solvent A was water containing 0.1% formic acid, and solvent B was 100% acetonitrile containing 0.1% formic acid. Peptides were separated as follows: solvent A was maintained at 2% followed by an increase to 7.5% in 5 min, 7.5% to 25% in 45 min, 25% to 45% in 15 min, 45% to 80% in 0.1 min, maintained at 80% for 10 min, followed by a decrease to 2% in 0.1 min and equilibration at 2% for 10 min. The Orbitrap Fusion was operated in positive-ion data-dependent mode and precursor ions (MS1) were detected in the Orbitrap with a nominal resolution of 120 000 at 200 *m*/*z*. An automatic gain control (AGC) target of 5  ×  10^5^ and an ion injection time of 50 ms were used. The most intense ions above a threshold of 5 × 10^3^ were selected for high-energy collision dissociation at a normalized collision energy of 32.5%. The fragmented ions were analysed in the Orbitrap (MS2) at a resolution of 15 000 at 200 *m*/*z*. The AGC target was set to 1  ×  10^4^ and a 45 ms injection time was allowed during MS2 analysis. The number of MS2 events between MS1 scans was determined on the fly to maintain a 3 s fixed duty cycle. Dynamic exclusion of ions within a ±10 ppm *m*/*z* window was implemented using a 30 s exclusion duration. An electrospray voltage of 2.0 kV and capillary temperature of 275°C, with no sheath and auxiliary gas flow, were used.

### Processing of LC–MS/MS data

A custom-made *M. tuberculosis* reference proteome was generated as described in the ‘DNA extraction and whole genome sequencing analysis’ section, specifically by addition of the putative chimeras to the reference proteome. Amino acid sequences representing potential gene fusions were added to the reference proteome (UniProt accession: UP000001584) from *M. tuberculosis* H37Rv (downloaded from UniProt, 10 April 2018). This custom database was used for all mass spectrometry searches and contained a total of 4002 entries, which include the potential gene fusions. MaxQuant (version 1.6.3.4) was used to analyse raw files obtained from LC–MS/MS. The Andromeda search algorithm, integrated in MaxQuant, was used for peptide and protein identification, using default parameters (38,39). Carbidomethylation of cysteine was chosen as fixed modification and oxidation of methionine as well as N-terminal acetylation was chosen as variable modification. Enzyme specificity was set as trypsin/P and a maximum of two missed cleavages were allowed.

### PPE38 C-terminal domain cloning, expression and purification

Genomic DNA was extracted from *M. tuberculosis* CDC1551 as described earlier and the region encoding the PPE38 C-terminal target was amplified using primers PPE38_AB_F: CCG CGA CGT GCT AGC ATG GCG GTG GAG GGG GTG CCG GC and PPE38_AB_R: TCA CAG GTC AAG CTT CTA CGC CGA CAT CCC CGC ACC CA with Phusion hotstart 2× master mix polymerase (NEB, MA, USA). The resulting amplicon was cloned into pIBA (40), using an In-Fusion cloning reaction as described by the manufacturer (Takara Bio, Japan), in *E. coli* Top10F′ cells. Transformed cells were cultured on LB agar supplemented with ampicillin (100 μg/ml). The presence of the *ppe38* insert was verified by restriction digest and Sanger sequencing. For recombinant protein expression, transformed *E. coli* Top10F′ was cultured in LB broth supplemented with 100 μg/ml ampicillin. Expression was induced with 0.2 μg/ml anhydrotetracycline (Sigma-Aldrich, MO, USA) at 37°C with shaking at 200 rpm when *E. coli* cultures reached an OD_600_ of 0.3 for 2 h or until an OD_600_ of 1.5. Cultures at OD_600_ of 1.5 were subsequently harvested by centrifugation (14 000 rpm, 10 min, 4°C) and suspended in ice-cold lysis buffer (10 mM Tris–HCl, pH 8.0, 1 mM EDTA, 5 μg/ml lysozyme), followed by incubation at 37°C for 1 h. Cells were further disrupted by sonication (Branson Sonifier 250) and the lysate was centrifuged (12 000 rpm, 15 min, 4°C) to sediment inclusion bodies. Contaminating factors were removed by resuspending the pellet in sonication buffer (10 mM Tris–HCl, pH 8, 1 mM EDTA) and disrupted by sonication, followed by addition of an equal volume of Triton wash buffer (10 mM Tris–HCl, pH 8.0, 1 mM EDTA, 2% Triton X-100) and incubation for 1 h at room temperature. Inclusion bodies were collected by centrifugation (12 000 rpm, 15 min) and suspended in sonication buffer followed by another round of sonication. An equal volume of urea wash buffer (10 mM Tris–HCl, pH 8.0, 2 M urea) was added followed by 1 h incubation at 37°C. After incubation, the inclusion bodies were harvested by centrifugation (123 000 rpm, 15 min) followed by resuspension (sonication buffer) and sonication. An equal volume of high-salt wash buffer (10 mM Tris–HCl, pH 8.0, 2 M NaCl) was added to the resulting suspension and inclusion bodies were harvested by centrifugation (12 000 rpm, 25 min). Another round of sonication and centrifugation followed this step and the final inclusion body-containing suspension was stored in PBS containing 15% glycerol.

Inclusion bodies were separated by 10% SDS-PAGE (1× TGS buffer, 100 V, 1 h) and visualized with Coomassie Brilliant Blue. These inclusion bodies were used for immunization in rabbits performed by Innovagen (Sweden) as detailed in their custom polyclonal rabbit IgG service. Briefly, initial immunization was done with the inclusion body suspension containing the PPE38 antigen with Freund’s complete adjuvant. This was followed by booster immunizations at weeks 3, 5, 8 and 11 with Freund’s incomplete adjuvant. Immune serum used for this study was collected at 13 weeks after immunization. The resulting anti-serum from the immunized rabbit was used for western blotting.

### Western blotting

Concentrated supernatants from *M. tuberculosis* CDC1551, *Δppe38–71*, the *ppe38–71* complemented strain, S507, S3651, S1453, S3760, S4437, S3839, S5218, H37Rv, S4570, S1116, S2701 and S2135 were separated by SDS-PAGE (12% resolving gel, 100 V, 1 h) and transferred to a nitrocellulose membrane. The membrane was blocked with 1% milk powder (Sigma-Aldrich, MO, USA) for 1 h and probed with either mouse monoclonal α-PGRS (1:5000) (41) and α-EsxA (1:500) (42) or rabbit polyclonal α-PPE38 (1:1000) (this study) overnight at 4°C. Primary antibodies were removed by washing with Tris-buffered saline supplemented with Tween (20 mM Tris–HCl, pH 7.5, 150 mM NaCl, 0.1% Tween 20) followed by probing with either goat anti-mouse or goat anti-rabbit horse radish peroxidase conjugated secondary antibody for 1 h. Probed nitrocellulose membranes were visualized using a ChemiDoc Gel Imaging System (Bio-Rad, CA, USA). When required, membranes were stripped using mild stripping buffer (1.5% glycine, 0.1% SDS, 1% Tween 20, pH 2.2).

### Polymerase chain reaction and Sanger sequencing

Polymerase chain reaction of the Rv2623–Rv2628 region was performed using Phusion polymerase (New England Biolabs, MA, USA) as indicated by the manufacturer with the addition of 2% dimethyl sulfoxide. Products from *M. tuberculosis* H37Rv and clinical isolate S507 were sequenced by capillary electrophoresis to identify and confirm the fusion junction using the following primers: Rv2623_F: CCA TTG TCG CGC ACA AAC and Rv2628_R: GTG GCA TGG CCA TGT CTT CTA.

### Statistical analysis

**Figure 1.**
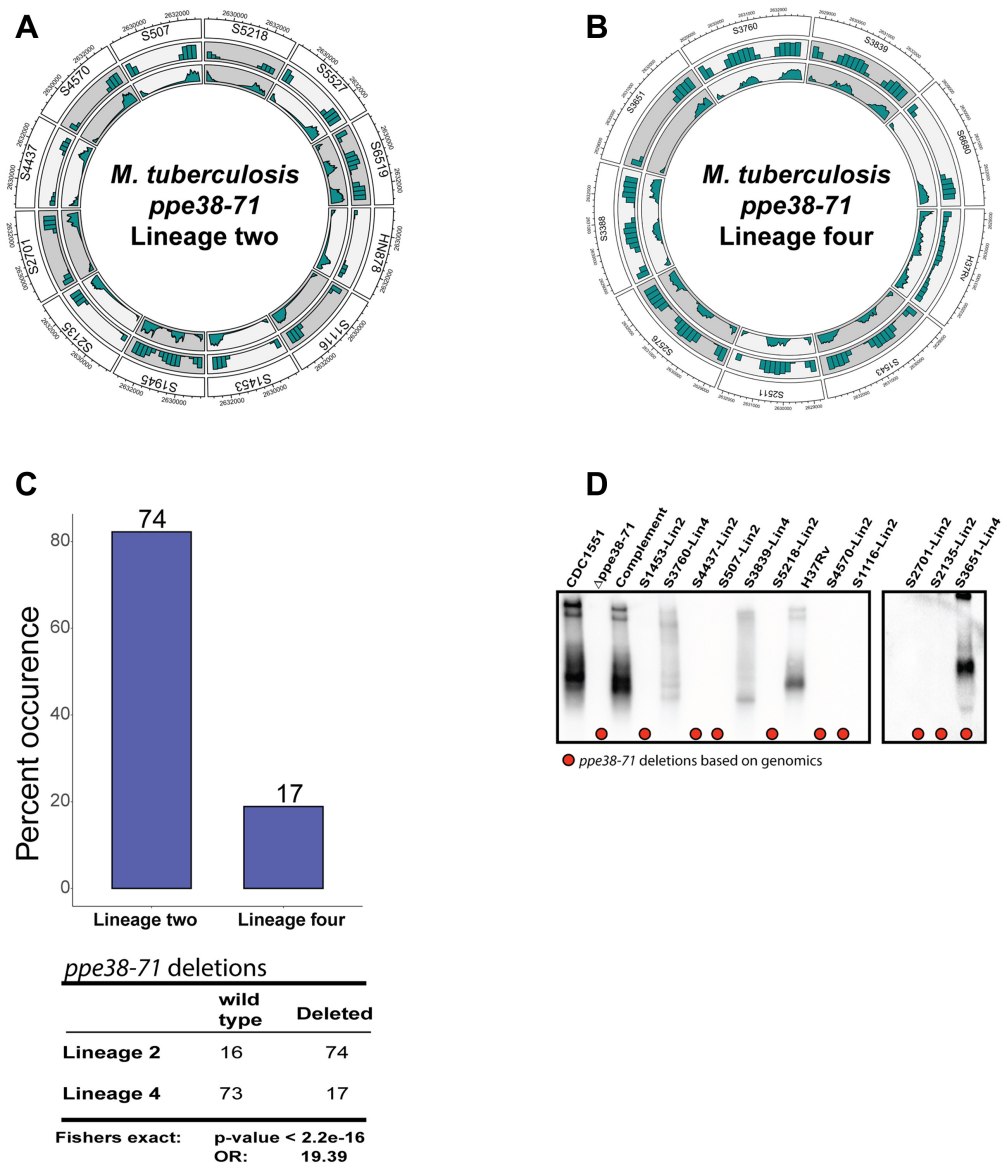
Deletions in the *ppe38–71* operon are more prevalent in *M. tuberculosis* lineage 2 isolates. Deletions are represented as Circos plots displaying whole genome sequence alignment by genomic coordinates (outer track), strain (first track), read density (middle track) and average coverage (inner track) for clinical isolates of *M. tuberculosis* representing (**A**) lineage 2 and (**B**) lineage 4. Averages were determined per strain and only within the genomic coordinates spanning the deletion. (**C**) Occurrence of *ppe38–71* deletion as a percent calculated from each subpopulation of lineage 2 and lineage 4 representing 90 clinical isolates, respectively. Deletions were determined by inspecting coverage in the *ppe38–71* operon. (**D**) Western blot of cell-free supernatants obtained from *M. tuberculosis* clinical isolates, *M. tuberculosis* CDC1551 and control strains targeting PE-PGRS proteins. Red dots indicate clinical isolates that were predicted by whole genome sequencing to have a *ppe38–71* deletion.

All statistical tests performed downstream using Delly and Lumpy-SV outputs were implemented in the R programming language version 3.6.2. The data presented in Figure 1A were analysed as a contingency table using Fisher’s exact test with a *P*-value cut-off set at 0.05.

## RESULTS

### *ppe38–71* deletions are more prevalent in lineage 2 isolates of *M. tuberculosis*

A previous study in our group has shown that deletions within the *ppe38–71* locus in certain strains of *M. tuberculosis* can result in a block of PE-PGRS secretion and are associated with increased virulence (13). An earlier study already indicated that the *ppe38–71* operon is prone to recombination with multiple variations (43). However, because of the highly repetitive nature and high GC content of this region, these deletions are difficult to identify. In this study, we further investigated the genetic relationship between *ppe38–71* deletions in various lineages of *M. tuberculosis* and the effect of this mutation on PE-PGRS protein secretion. Twenty-one well-characterized and genetically diverse clinical isolates representing lineage 2 and lineage 4 were used as the initial screening group (Supplementary Figure S1A in Supplementary File S1, Supplementary Table S1).

A custom Illumina data analysis pipeline was constructed, which used two split read callers, a coverage-based approach and a targeted approach, to find both known and unknown deletions (Supplementary Figure S2 in Supplementary File S1). To address the variability within the region, two genes that fall between *ppe38* and *ppe71* (*mt2420* and *mt2421*) were examined. These genes are unaffected by the insertions of IS6110 and reads mapping to this region were therefore used as an indicator for a full *ppe38–71* operon (Supplementary Figure S3 in Supplementary File S1). Using these criteria, *ppe38–71* deletions were detected in both lineage 2 and lineage 4. The majority of the lineage 2 strains (Figure 1A) had *ppe38–71* deletions, while the converse was observed for lineage 4 strains (Figure 1B). This suggested a greater prevalence of this deletion, and by association a predicted lack of PE-PGRS secretion, in lineage 2 isolates. However, the members of both lineages are sampled within the same local geographical region and thus provide a bias within each distinct lineage. To detect whether this deletion was indeed more prominent in *M. tuberculosis* lineage 2 as isolates compared to lineage 4, 90 members from each of the two lineages were further screened for a total of 180 genomes (Supplementary Figures S1B and S4A1–A6 in Supplementary File S1). Using the same metric to detect this deletion as described earlier, we found a 74% occurrence of the *ppe38–71* deletion in lineage 2 and a 17% occurrence in lineage 4, thus indicating a significantly higher prevalence (*P*-value <2.2e−16) of *ppe38–71* deletions in the lineage 2 isolates (Figure 1C).

Previous publications demonstrated that breakpoints occur within *ppe38* and *ppe71* coding regions, effectively truncating the coding sequences of these genes and abolishing PE-PGRS secretion (13,43). This phenotype was tested in selected lineage 2 and lineage 4 clinical isolates by immunoblotting with a monoclonal antibody that specifically recognizes repeat domains (44). No PE-PGRS secretion was observed in members of lineage 2 that also had a *ppe38–71* deletion, as determined by whole genome sequencing (Figure 1D). Interestingly, PE-PGRS secretion was observed in S3651, which also has a *ppe38–71* deletion as determined by whole genome sequencing (Figure 1D).

In the ancestral operon, the *ppe38* and *ppe71* genes are nearly identical copies of one another separated by two *esx* genes (43). It has been suggested that deletion of the intervening sequence can result in a fusion protein (43). We thus reasoned that the presence of PE-PGRS proteins in S3651 culture supernatants could be due to the internal deletion causing the open reading frames of *ppe38* and *ppe71* to form a chimeric protein with a similar function to PPE38.

### *ppe38–71* deletion creates a natural chimera in clinical isolate S3651

**Figure 2.**
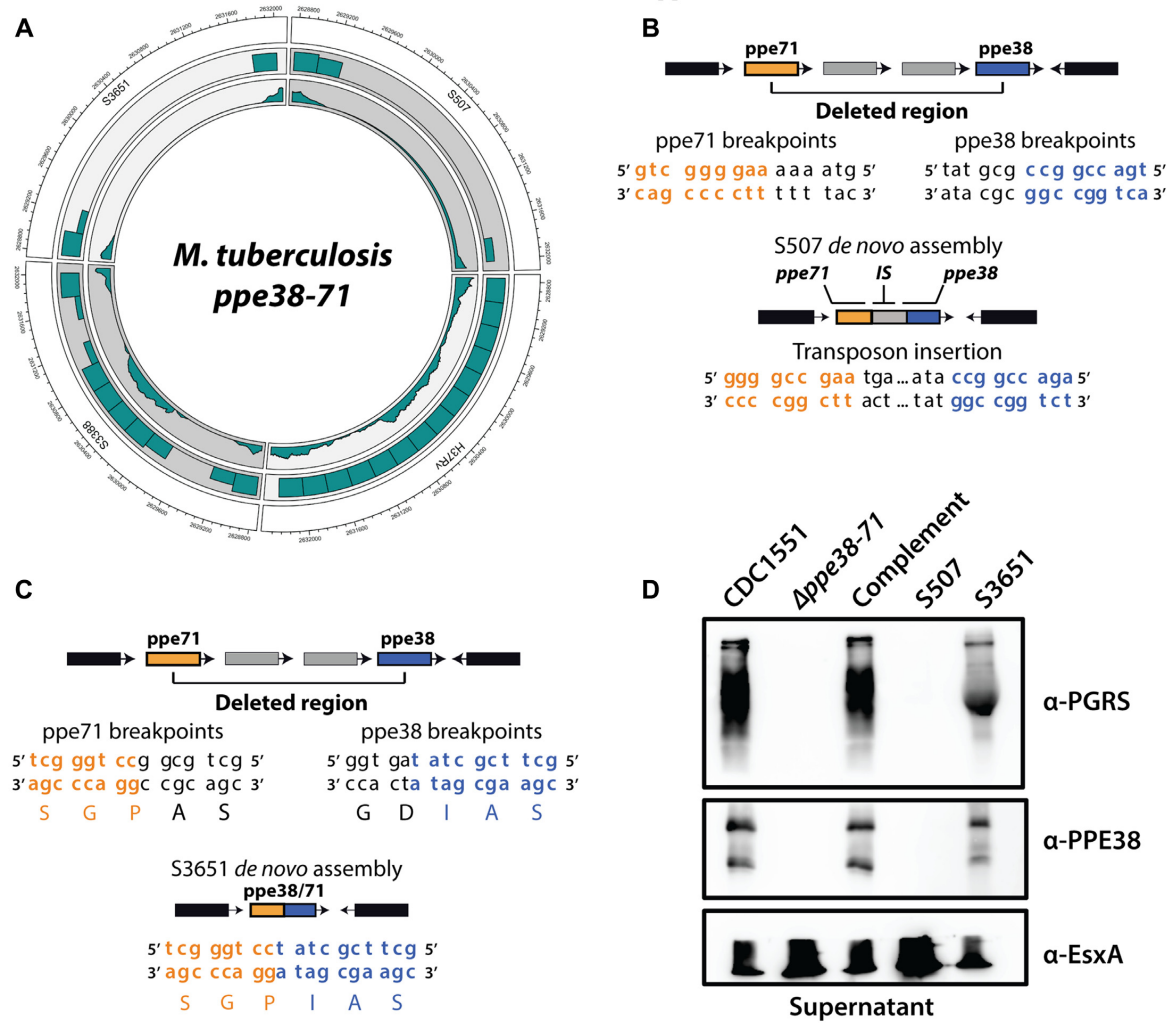
*Mycobacterium tuberculosis* lineage 4 strain with a *ppe38–71* deletion produces a functional chimeric protein. (**A**) Specific clinical isolates used for further investigation of the *ppe38–71* operon. *M. tuberculosis* H37Rv was used as a control for the NGS and aligned to CDC1551; full-length *ppe38–71* is detected in contrast to the published reference. S3651 and S507 represent clinical isolates with a *ppe38* deletion and S3388 represents a clinical isolate without deletion. (**B**) Schematic representation of targeted *de novo* assembly and contig ordering of S507 indicating a transposon insertion between *ppe71* and *ppe38*, thereby disrupting the *ppe71* reading frame. (**C**) Schematic representation of targeted *de novo* assembly in the *ppe38–71* operon of S3651. No transposon insertion was found and the reading frame of *ppe71* is intact causing a gene fusion. (**D**) Western blot of *CDC1551* reference, *Δppe38–71, ppe38–*71 complemented strain, S507 and S3651 cell-free supernatant probed for PE-PGRS proteins, PPE38 and ESAT-6 as the loading control. S3651 secreted PE-PGRS proteins and expressed PPE38 similarly to the wild type.

We investigated different configurations of the *ppe38–71* operon in more detail to determine whether a functional *ppe38/71* chimeric protein was indeed present in the lineage 4 isolate, S3651. For this, we compared S3651 to S507 as an example of a lineage 2 isolate with a *ppe38–71* deletion, S3388 as an example of a lineage 4 isolate with a full-length *ppe38–71* operon and *M. tuberculosis* H37Rv that also has a full-length *ppe38–71* operon (Figure 2A).

As transposon insertions have previously been found in the *ppe38–71* operon (13), we reasoned that the presence of a transposon will disrupt the open reading frames in cases where PE-PGRS secretion is abolished. *De novo* assemblies were used to target the *ppe38–71* operon in S507 and S3651, which represent PE-PGRS secretion negative and positive phenotypes, respectively. This was accomplished by extracting aligned reads for ∼2000 bp upstream and downstream of the *ppe38* and *ppe71* breakpoints. Targeted *de novo* assembly revealed that S507 (lineage 2) has an insertion sequence present between *ppe38* and *ppe71*, which disrupts the reading frames (Figure 2B, Supplementary Figure S5A in Supplementary File S1). However, *de novo* assemblies of S3651 indicated breakpoints causing the open reading frames of *ppe38* and *ppe71* to fuse and create a *ppe38/71* gene fusion (Figure 2C). The *ppe38/71* fusion is created through an out-of-frame deletion spanning the *ppe38* and *ppe71* operon, situated on the reverse strand, with breakpoints occurring within the open reading frame of each respective protein. Specifically, a cytosine–cytosine pair remaining on the *ppe71* breakpoint combines with a thymine at the *ppe38* breakpoint generating a cytosine–cytosine–thymine codon (Supplementary Figure S5B in Supplementary File S1). This recombination reinstates the proline originally encoded by cytosine–cytosine–guanine in *ppe71*, thereby creating the amino acid sequence S G P I A S reading from the N-terminal of PPE71.

Taking into account the secretion of PE-PGRS proteins found in S3651 (Figure 1D) as well as the predicted reformation of a distinct, yet functional equivalent of *ppe38* in S3651 (Figure 2C), we reasoned that this gene fusion is likely responsible for the PE-PGRS secretion phenotype. To detect expression of *ppe38*, we generated a rabbit polyclonal antibody against the C-terminal domain of *M. tuberculosis* PPE38, which was used to immunize rabbits. Rabbit anti-serum was first tested for reactivity against PPE38 (Supplementary Figure S6A and B in Supplementary File S1). Western blots against *M. tuberculosis* supernatants revealed the presence of PPE38 epitopes in *M. tuberculosis* CDC1551, the *ppe38–71* complemented strain and *M. tuberculosis* S3651. As expected, PPE38 was not detected in S507 and *Δppe38–71* strains (Figure 2D). Interestingly, two bands were detected when probing for PPE38, and both bands were absent in both S507 and *M. tuberculosis Δppe38–71* (Figure 1D, Supplementary Figure S6B in Supplementary File S1). This may occur due to cleavage of PPE38 through a protease, possibly PecA, which has been shown to cleave PE-PGRS proteins (45). Staining with anti-serum directed against PE-PGRS proteins resulted in a similar pattern, providing evidence that the production of PPE38 and secretion of PE-PGRS are linked and that the chimera formed in S3651 is associated with the PE-PGRS secretion.

### Detecting multigene deletions in *M. tuberculosis* clinical isolates

The presence of functional gene fusions in *M. tuberculosis* is an intriguing finding, which in the absence of horizontal gene transfer may provide an alternate method for evolutionary adaptation. To find additional chimeric proteins that have the potential to form gene fusions, we searched for genomic features likely to result in a gene fusion event. This was automated and coupled with *de novo* assembly, which was used to generate consensus sequences surrounding multigene deletions (Supplementary Figure S7 in Supplementary File S1). This approach served as a first-pass method to identify multigene deletions present within coding sequences and is thus a starting point to identify potential gene fusions. This algorithm was incorporated into our existing software and used to screen clinical *M. tuberculosis* isolates for possible gene fusions.

**Figure 3.**
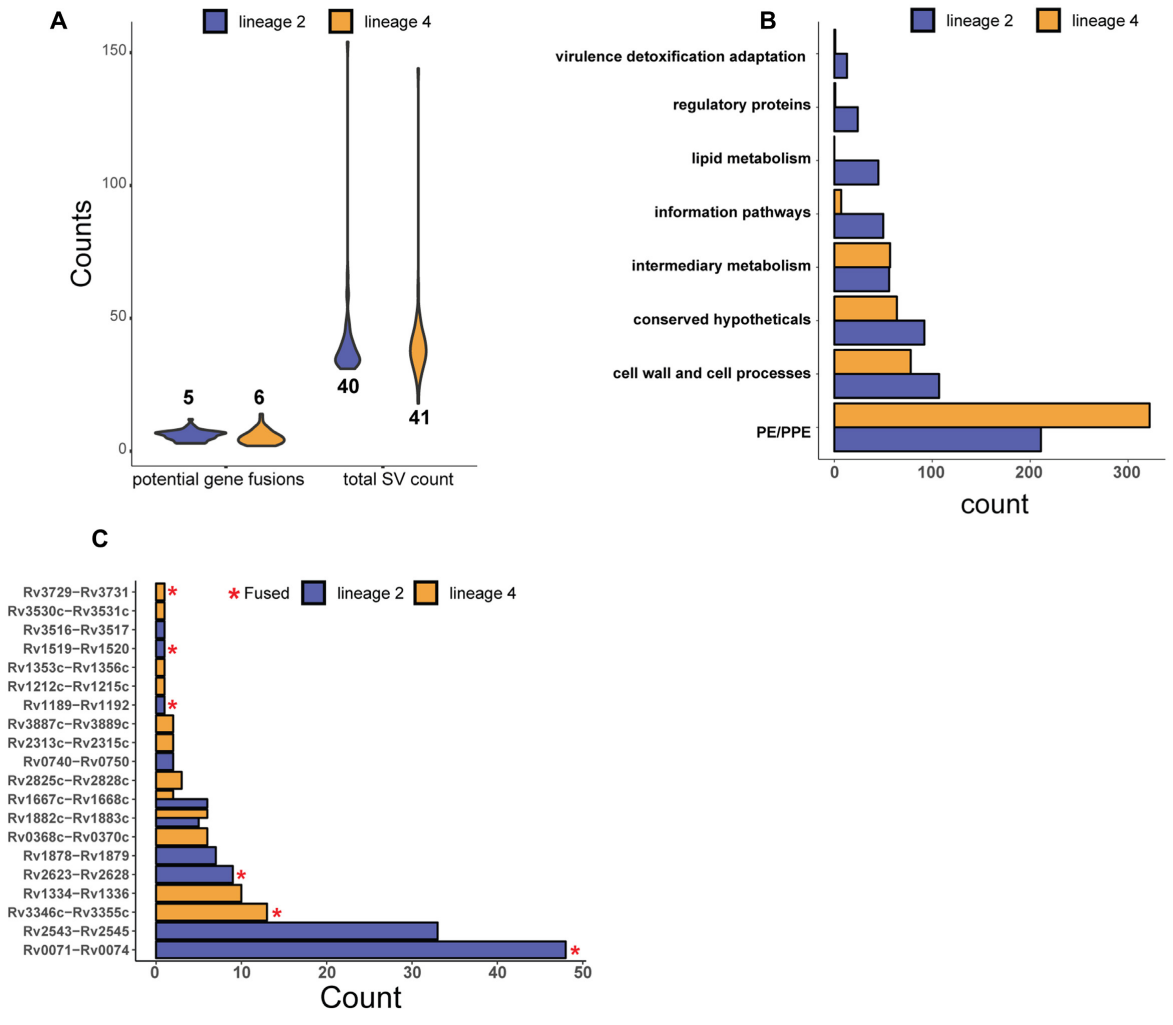
Systematic detection of potential fusion events from *M. tuberculosis* large sequence polymorphisms. (**A**) The distribution of multigene deletions that fall within an open reading frame and have the same orientation as predicted by our software compared to the distribution of SVs found across 180 isolates of lineage 2 and lineage 4. The numbers on the graph represent the mean of the distribution. (**B**) Most abundant annotations associated with potential gene fusions across all clinical isolates and separated by lineage. Annotation terms were sourced from the Mycobrowser functional categories. PE/PPE proteins constituted the majority of identifications associated with gene fusions. Potential gene fusions falling in this category were removed from further consideration as alignment failures in these areas are highly prevalent. (**C**) Occurrence of specific multigene deletions that fall within open reading frames. Each of these were manually inspected for closed reading frames and annotated as either fused or truncated.

We screened the genomes of the same 180 clinical isolates mentioned earlier for multigene deletions that can form potential gene fusions and compared this to all the SVs detected. The occurrence of gene fusions represented a low proportion of the total structural variation (Figure 3A). Furthermore, the SV count had more variability across the two lineages with some strains containing >100 SVs. This was observed in both lineages; however, this increase in the total number of SVs did not affect the distribution of potential gene fusions (Figure 3A). Thus, formation of gene fusions seems to be a rare phenomenon likely due to the inherent randomness in genetic variation and the precise breakpoints required to maintain a reading frame. Once this process concludes and a chimeric protein is formed, the new protein has to be favoured by natural selection to allow for propagation of the new feature within the population. It therefore stands to reason that formation of fused genetic elements that yield proteins is less likely to occur compared to pseudogene formation, full gene formation or out-of-frame deletions. The highest occurrence of multigene deletions was found in genes encoding PE/PPE proteins; however, due to problematic read alignment in these regions (very high GC content of >75% and multiple repeats), it is difficult to determine with confidence that these are indeed true gene fusion events (Figure 3B). We therefore discarded multigene deletions occurring within genes annotated as PE/PPE proteins during further analysis. This resulted in the identification of 21 multigene deletions, 2 of which were found within both lineages. Multigene deletions that resulted in fused open reading frames from the genomic data were further manually inspected by six-frame translation of the fusion gene. If the six-frame translation indicated a single protein with domains from both parent genes, we annotated this as fused (Figure 3C). We could detect the RD^RIO^ deletion that forms a fused reading frame in Rv3346c/55c (Figure 3C) (46). However, the most prevalent multigene deletion that has fused open reading frames in our sample set was represented by the RD105 deletion (*Rv0071/74*) that occurs in lineage 2 isolates (47) (Figure 3C).

### Genetic evidence for gene fusions in mycobacteria

**Figure 4.**
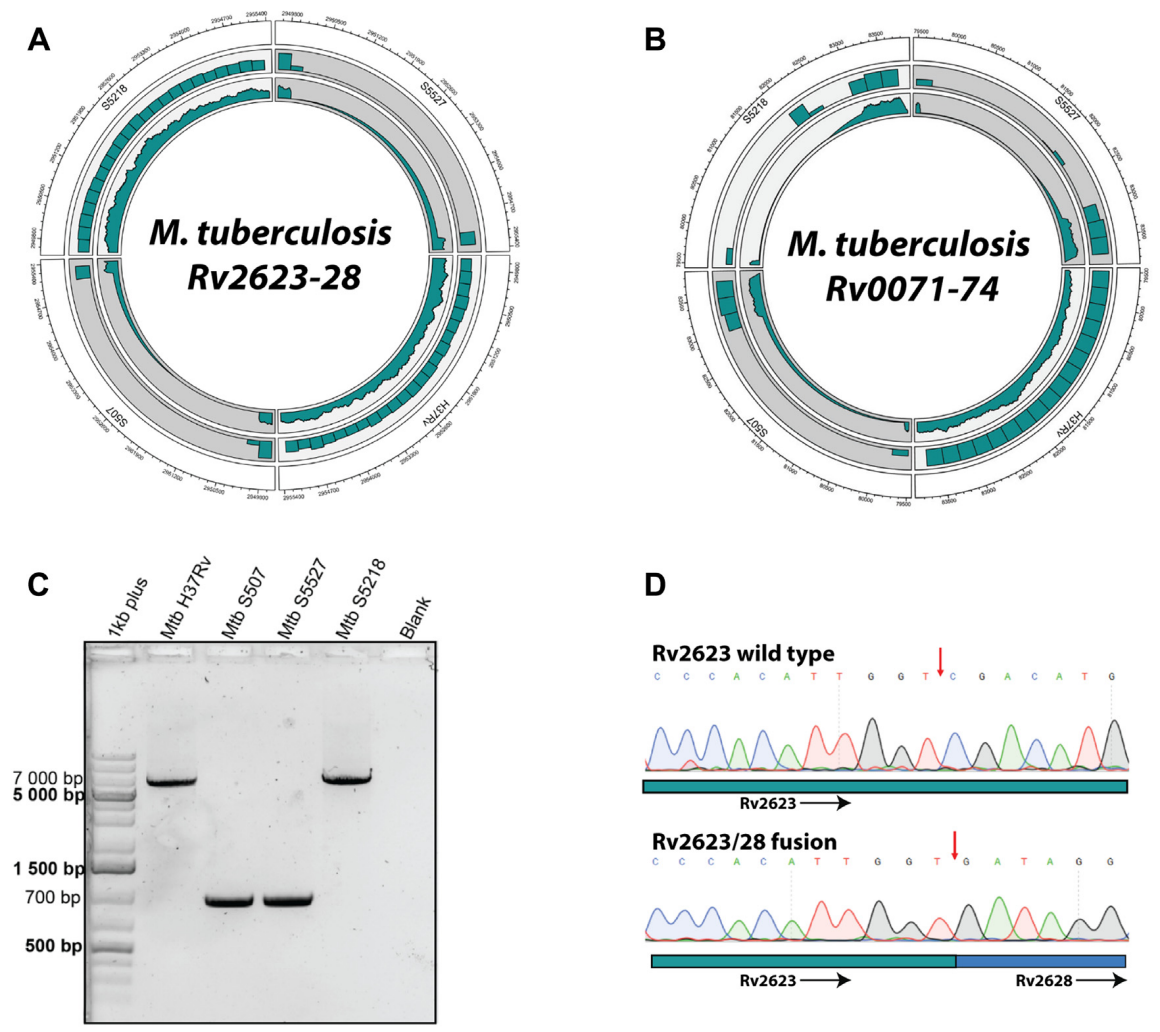
*Rv2623/28* and *Rv0071/74* are gene fusions that have formed as a result of large deletions. (**A**) Circos plot depicting the genomic region of *Rv2623–Rv2628* (outer track) from clinical isolates S5218, S5527, S507 and H37Rv. Middle and inner tracks display read density in the region and average coverage, respectively. (**B**) Circos plot of *Rv0071–Rv0074* (outer track) as well as the read density (middle track) and average coverage (inner track) in the region. (**C**) Polymerase chain reaction of wild-type and deleted Rv2623–Rv2628 regions. (**D**) Chromatograms from capillary electrophoresis displaying the deletion breakpoints (red arrow) from clinical isolate S5218 (wild-type Rv2623) and S507 (Rv2623/Rv2628 fusion protein). In the Circos tracks, *M. tuberculosis H37Rv* is representative of the reference genotype.

Three candidate gene fusions were observed at relatively high frequency within the 180 *M. tuberculosis* genomes, compared to the other gene fusions (Figure 3C). We opted to focus on *Rv0071/74* and *Rv2623/28* for further investigation, while *Rv3346c/55c* was disregarded as this specific deletion was not found within the 21 well-characterized clinical isolates and has been described earlier (46). The *Rv2623–Rv2628* genomic region is part of the *M. tuberculosis* dormancy regulon and has not yet been reported as deleted within clinical isolates of *M. tuberculosis*. This region is deleted in lineage 2 members, S507 and S5527, where the deletion completely removes the genes *Rv2624–Rv2627* and truncates *Rv2623* at the 5′ end, coding for the C-terminal domain, and *Rv2628* at the 3′ end (Figure 4A). The RD105 deletion was also detected as a gene fusion using our pipeline and recently shown to indeed form a chimeric protein (Figure 4B) (18). This deletion is prevalent in the lineage 2 isolates and is used as a marker for sub-lineage speciation within *M. tuberculosis* (47). The protein encoded by *Rv0071* is annotated as a potential maturase and the *Rv0074* gene product is of unknown function and is localized in the membrane of *M. tuberculosis*, as determined by mass spectrometry analysis (48).

The Rv2623/28 putative gene fusion, identified by our method, has not yet been reported as a gene fusion or indeed a deletion in the mycobacteria. We therefore characterized this feature further by verifying the presence of this deletion as well as the specific breakpoints using polymerase chain reaction and capillary electrophoresis sequencing. First, the deletion was confirmed in S507 and S5527 identifying a band at the 700 bp range using primers flanking *Rv2623* and *Rv2628*, compared to a band of ∼7000 bp in S5218 and H37Rv that have an intact operon (Figure 4C). Next, the 700 and ∼7000 bp bands corresponding to S507 and S5218 were sequenced, respectively. The base pair sequence corresponding to wild-type *Rv2623* and the *Rv2623/28* fusion was discernible and corresponded to the sequence prediction from our *de novo* assemblies of this genomic region (Figure 4D). Based on these observations, the Rv2623/28 remains intact and should transcribe a hybrid protein under the Rv2623 promoter.

### Gene fusions form chimeric proteins

*Mycobacterium tuberculosis* follows a reductive evolutionary path and consequently has a number of pseudogenes (49). Although the candidates found in our genetic screens may have the genotypic characteristics of a chimeric protein, these may not lead directly to proteins. The genetic features may thus be present within the genome, not as functional chimeras but rather as pseudogenes.

We used mass spectrometry analysis to investigate whether the putative gene fusions found in our genetic screens are expressed by *M. tuberculosis* to form stable chimeric proteins. To identify chimeric proteins, the translated sequences obtained from the *de novo* assemblies of *ppe38/71;*Rv2623/28 and Rv0071/74 were added to the *M. tuberculosis* H37Rv protein database (UP000001584). The *pks15/1* gene is found in W-Beijing strains of *M. tuberculosis* and is comprised of fused *pks15* and *pks1* genes (22,50). This gene was not detected in our genetic screens of W-Beijing strains, likely due to the close proximity of the breakpoints causing discordant read pairs to be missed. We therefore added this chimeric protein sequence as well, as it has previously been shown to combine in a manner similar to what we predict for gene fusions (22,50). This database was used to search tandem mass spectra from S507, S5527 and S5218 generated in this study as well as S3651, which is publicly available and generated from a previous study (19).

MS-Digest, a tool available with the ProteinProspector software (51), was used to model the tryptic peptides of the potential chimeric proteins surrounding the expected fusion junctions of ppe38*–*71;Rv2623/8, Rv0071/74 and Pks15/1 as well as their wild-type counterparts. Translation of the *de novo* assembly of the *ppe38/71* operon predicts the fusion junction to have the amino acid sequence S G P I A S (Book S1 in Supplementary File S2). The fusion junction amino acid sequence corresponding to V I G R is expected if the Rv2623/8 fusion is present in S507 and S5527, while D M S K is expected in wild-type S5218 (Book S2 in Supplementary File S2). If *Rv0071/74* is produced in the lineage 2 strains, the fusion junction V V G V G R should be detected (Book S3 in Supplementary File S2). Lastly, if Pks15/1 is present, an amino acid sequence corresponding to V P W V I S A R is expected (Book S4 in Supplementary File S2).

**Figure 5.**
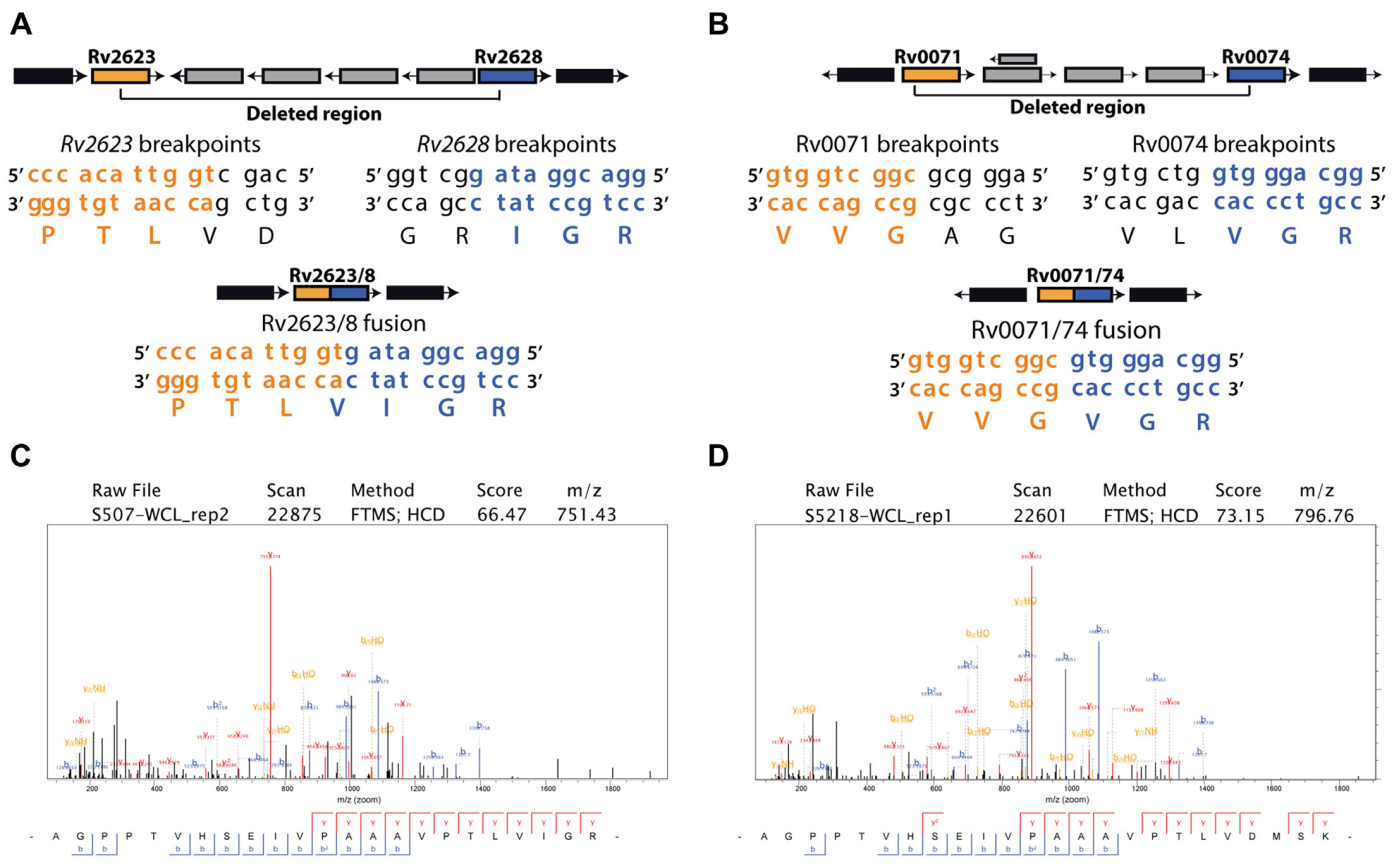
Targeted *de novo* assemblies and tandem mass spectrometry identify Rv2623/28 as chimeric protein. (**A**) Schematic illustration of *Rv2623–Rv2628de novo* assembly and contig ordering from clinical isolate S507. (**B**) Schematic illustration of *de novo* assembly and contig ordering from S507 displaying the deletion region and in-frame translation of *Rv0071/74*. Black indicates out-of-bounds genes and grey indicates deleted genes. Tandem mass spectra of peptides representing (**C**) the Rv2623/28 fusion junction from S507 and (**D**) the wild-type Rv2623 of S5218 in the same location. False discovery rate cut-off for assigning peptides was set at 0.01.

We used *de novo* assemblies to determine the fusion junctions of *Rv2623/28* and *Rv0071/74* genetically. The *Rv2623–Rv2628* deletion results in a new fusion gene with a restored reading frame (Figure 5A). The *Rv0071*–*Rv0074* deletion has an in-frame breakpoint at position 93 (gtc) of the *Rv0071* gene, with a codon for alanine (31st amino acid), as well as at position 289 (gtg) of *Rv0074* that codes for valine (97th amino acid), thus closing the frame to create a V V G V G R amino acid sequence (Figure 5B). This fusion candidate has recently been demonstrated to indeed encode for a functional chimeric protein, demonstrating a functional phenotype associated with this genotype (18). Next, we used our custom gene fusion database as a reference for tandem mass spectra searches to detect Rv2623/28 and Rv0071/74 in clinical isolates S507 and S5527 while using S5218 as a control. Tandem mass spectra from previously published S3651 were also searched by using the same approach to probe for *ppe38/71*. We detected the presence of a peptide that spans the Rv2623/28 fusion junction, thereby providing evidence for the expression of another chimeric protein in *M. tuberculosis* (Figure 5C). While Figure 5C represents the most confident peptide identification by the Andromeda search engine, in this specific peptide, fragmentation did not cover the fusion event located in the y1–y7 ion range. We found seven peptide spectrum matches of fragmentation across the fusion junction, thereby supporting the identification of this peptide as spanning the fusion junction between Rv2623 and Rv2628 (Supplementary Figure S8A1–A7 in Supplementary File S1). In addition, we were able to detect peptides corresponding to wild-type Rv2623 in clinical isolate S5218 (Figure 5D). We could not detect a peptide corresponding to the wild-type Rv2623 protein in S507 or S5527, and likewise no fusion peptide was detected in S5218 (Supplementary Figure S8B in in Supplementary File S1). In addition, peptides spanning the fusion junction for Pks15/1, previously reported as an insertion, were also detected in our mass spectrometry analysis (Supplementary Figure S9 in Supplementary File S1) (22).

No peptides corresponding to PPE38/71 or Rv0071/74 fusion junctions were detected by mass spectrometry analysis. This may be due to several reasons such as low abundance, low coverage of the proteome or, in the case of Rv0071/74, a large amount of proline repeats across the fusion junction. Nevertheless, Rv0071/74 was recently shown to form a chimeric protein and to be involved in the remodelling of the bacterial cell wall and has been associated with increased drug resistance (18). Furthermore, using polyclonal anti-serum directed against PPE38, we were able to show that a chimeric protein was produced in strains containing the PPE38/71 fusion. Taken together, it is evident that gene fusions not only are present within *M. tuberculosis*, but can also encode functional proteins.

## DISCUSSION

In this study, we present a computational method to discover chimeric proteins using large-scale omics data. By using unique features found in individual genomes in a proteogenomics approach, structures such as chimeric proteins can be identified in the proteomes of clinical *M. tuberculosis* isolates. While similar methodology has been implemented in other biological fields, such as cancer research (52), studies have not yet been conducted in a high-throughput fashion in bacteria. Using the same methodology proposed here, the same approach can be broadly applied to other bacteria as genomics and proteomics data become more prevalent.

It has been hypothesized that *M. tuberculosis* does not undergo horizontal gene transfer (11). This imposes a significant limitation on the evolutionary capabilities of the bacilli, especially when it is faced with constant evolutionary pressure due to the harsh environment of the phagosome (53). Proteins that have two distinct domains likely arose from two ancestral genes through gene fusion formation. These can be detected by comparative genomics between two or more related organisms. Gene fusion formation compresses the coding potential of the genome by creating multifunctional proteins through the combination of domains (54–56). This is especially effective if there is a clear evolutionary link between multiple species from which gene fusions can be found using tools such as MosaicFinder, DomainTeam or machine learning approaches (57–59). Therefore, the formation of gene fusions could provide interesting insights into the functional evolutionary biology of *M. tuberculosis*. This is especially useful under restrictive environments where reductive evolution is present (4). This is of importance as the bulk of studies mainly focus on single nucleotide variants and their functional role, while larger structural genomic variation is used for the purpose of strain typing (60,61). Interestingly, these large deletions are optimal for differentiation between lineages of *M. tuberculosis* as they are highly conserved according to a geographical origin (62). This is indicative of restrictive divergent evolution, where deletions occur frequently as a function of intracellular lifestyle yet are selected for by variable environmental conditions. The *ppe38–71* operon is a hypervariable region that arises due to the prevalence of transposon insertions (43). Therefore, the deletion can occur in both lineages and is not associated with a single event. However, the specific breaks in this region can have seemingly different consequences. Apart from loss of function, as a result of large-scale deletions, we demonstrate that these deletions can result in the formation of detectable chimeric proteins, which in turn likely has functional consequences. We could demonstrate such functionality for the *ppe38/71* fusion protein in the form of PE-PGRS secretion. Previous reports have shed light on the increased ability of lineage 2 *M. tuberculosis* to transmit and cause disease (63,64). As the lack of *ppe38–71* is also associated with increased virulence (13), it is likely compounding and contributing to the increased disease-causing capability observed in lineage 2 isolates (13). Furthermore, others have demonstrated functionality for Rv0071/74 (18), Rv3346c/55c (46) and Pks15/1 (22) chimeric proteins as well as the impact of large deletions on increased virulence (65). *Mycobacterium tuberculosis* strains can be grouped into sub-lineages by occurrence of large deletions that remove multiple genes in the process. This has the clear effect of abolishing the functions of the genes that have been lost, but also as seen in the case of RD107 and RD^RIO^ can form new coding sequences in the process. Contrary to the *ppe38–71* deletions, formation of the gene fusions is likely a result of selection and expanded to create sub-lineages and thus form a monophyletic group.

A new chimeric protein candidate, Rv2623/28, was identified in this study. The parent proteins for this chimera have been associated with entrance into dormancy (66,67) and associated with latency (68). Overexpression of Rv2623 causes a decrease in proliferation of *M. tuberculosis in vitro* and increased pathology in mice, thereby mediating entrance into dormancy (67). This protein is comprised of two ATP binding domains and the chimera Rv2623/28 has one of these domains followed by an Rv2628 N-terminus. If this domain remains stabilized with the Rv2628 N-terminus, it could result in a less pronounced inhibition of growth than reported for the full-length Rv2623 (67). Furthermore, the gene at the 3′ end of the deletion, *Rv2628*, is associated with latent tuberculosis infection as shown by a stronger cumulative interferon-gamma response towards Rv2628 antigens, compared to tuberculosis-positive individuals (69,70). The *Rv2623–Rv2628* gene fusion is therefore an interesting open reading frame potentially with aspects associated with both early- and late-stage dormancy.

Information on transcribed gene fusions can also be identified by RNA sequencing by using split read transcripts with multiple tools available for this purpose (71–73). A powerful approach presented by RNA sequencing is the use of *de novo* transcript assembly to mitigate the requirement of the reference sample; however, this suffers a penalty in the form of decreased accuracy (74). While RNA sequencing provides a powerful tool to identify gene fusions, the function of the resulting chimera is performed on the protein level. This is especially important as there have been reports on the poor correlation between RNA and protein content (75,76). This general discrepancy exists due to the complex mechanisms governing mRNA and protein regulation, both post-transcriptionally and post-translationally, and likely has a significant temporal component (77). Therefore, the combination of genomics and proteomics provides a powerful, yet underdeveloped, approach to high-throughput identification of expressed gene fusions. The combination of these technologies does, however, bring with it limitations associated with each respective platform. These limitations influence the detection power of a combined approach. From a genomics perspective, detection of gene fusions is influenced by the choice of a reference strain; this could, however, be mitigated by using a known ancestor or a metagenome approach by combining various related genomes in order to cover a broad range of genes (78). Long-read sequencing provides an alternative option to facilitate the process of resolving gene fusions in the genome. This can be used as a *de novo* assembly, reference-based assembly or a hybrid approach to genetically detect gene fusions in a similar fashion as the short reads used here. With longer reads, the exact breakpoints could be resolved with increased accuracy and thus result in the detection of more gene fusions. This is especially valuable as failure of alignments to pinpoint exact breakpoints can confound detection and either falsely call a gene fusion or miss potential gene fusions completely. Finally, a diverse library of sequences is necessary in order to effectively search for gene fusions using whole genome sequencing. In the Western Cape region of South Africa, where this study was conducted, the majority of *M. tuberculosis* strains are members of either lineage 2 or lineage 4. We therefore focused on these lineages of *M. tuberculosis* to act as our library and *M. tuberculosis* H37Rv as the reference, thus limiting the amount of gene fusions we could detect. As there are other lineages, characterized by large sequence polymorphisms, it is likely that there are more gene fusions to be identified.

The use of data-dependent tandem mass spectrometry analysis also limits the gene fusion detection ability. As the mass spectrometer only selects the ‘top *N*’ most intense precursor ions (79), a gene fusion would need to have a relatively high abundance to guarantee detection. In addition, proteins are not completely sequenced using shotgun proteomics; thus, fusion peptides that do not contain trypsin cleavage sites will be missed by this approach (80). As the fusion junction, and thus the fusion protein, is resolved by a single peptide, the likelihood for detection decreases as well. With arguably low genomic diversity in this cohort, where only two major lineages were screened, we still identified four candidate gene fusions using a genomics and proteomics approach. *Mycobacterium tuberculosis* is subject to reductive evolution, which is a marked characteristic of an intracellular lifestyle (1,81,82). With this limited and decreasing coding potential of *M. tuberculosis*, even rare occurrences of gene fusions and resulting chimeric proteins could result in significant phenotypic consequences. Some of the limitations to detect chimeric proteins in *M. tuberculosis* proteomes can, however, be overcome using a targeted proteomics approach to identify fusion junction peptides or using dedicated spectral libraries in conjunction with large-scale data-independent approaches such as SWATH-MS (83,84).

In this study, we focused on gene fusions and their detection. However, the use of similar methodology can be extended to other phenotypic features typically lost by reference-based assembly, such as identifying novel proteins from unmapped reads (19). Indeed, a significant number of tandem mass spectra remain unassigned after database searches (85), of which many display high-quality spectra (86). Spectra remain unassigned due to a multitude of reasons such as charge state, fully tryptic searches, post-translational or chemical modifications and errors in mass-to-charge measurements (51,87). It is also reasonable to assume that incomplete databases and unrepresented proteins also contribute to the large number of unassigned spectra. By expanding methodologies presented here and with further exploration of cross-platform omics technologies, previously hidden features can be extracted and provide a high-throughput approach to identifying novel features. In this study, *M. tuberculosis* was used; however, the same methodology can be implemented for other bacteria as well.

In conclusion, here we demonstrated that additional and often overlooked features of the genome contribute to the physiology of *M. tuberculosis*. These features provide a means to utilize vertical evolution to gain functionality in the absence of horizontal gene transfer. Further study into the effects of structural variation along with the mechanism that allows these features to translate to functional phenotypes could be important to understanding *M. tuberculosis* disease-causing capabilities.

## DATA AVAILABILITY

The gene fusion calling software is freely available under the GNU General Public License, version 3 (https://github.com/JamesGallant/Genomics). A Docker image of the software is also available at https://hub.docker.com/r/jamesgallant/pegasus. Accession numbers for genomes are available in Supplementary Table S1 and all the raw genomics data have been deposited in the European Nucleotide Archive under the project accession PRJEB36366. Accession numbers for proteomes and custom FASTA file are available on ProteomeXchange: PXD017298.

## Supplementary Material

lqaa033_Supplemental_Files
