# Supplementary material for: Identification of gene fusion events in Mycobacterium tuberculosis that encode chimeric proteins
Source: NAR Genom Bioinform. 2020 May 18;2(2):lqaa033. doi: 10.1093/nargab/lqaa033 (PMC7671302; doi:10.1093/nargab/lqaa033)
Supplement: lqaa033_Supplemental_Files [file lqaa033_supplemental_files.zip › additional file 1.pdf]

Additional file 1

Supplementary figure 1A

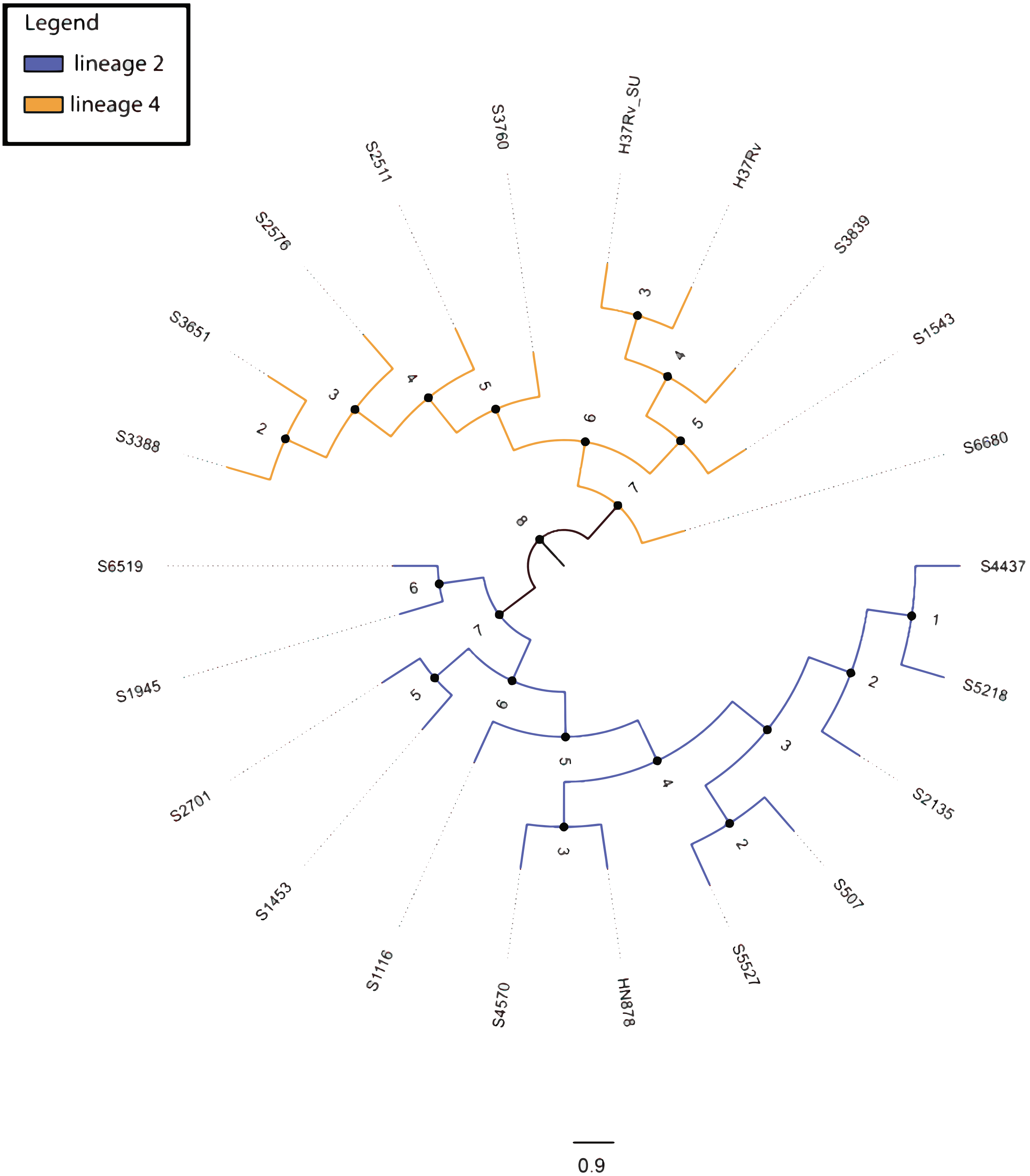

Figure S1A: Phylogenetic tree of *M. tuberculosis* clinical isolates. Phylogenetic tree represents the clinical isolates typed in supplementary table 1. The tree was constructed by aligning raw sequence data to *M. tuberculosis* H37Rv and identify single nucleotide variants from each isolate. The general time reversal model of nucleotide substitution was used to construct an axelerated maximum likelihood phylogeny of the isolates with 1000 bootstrap pseudo replicates. Positions with gaps or missing replicates were not considered in this analysis.

Supplementary figure 1B

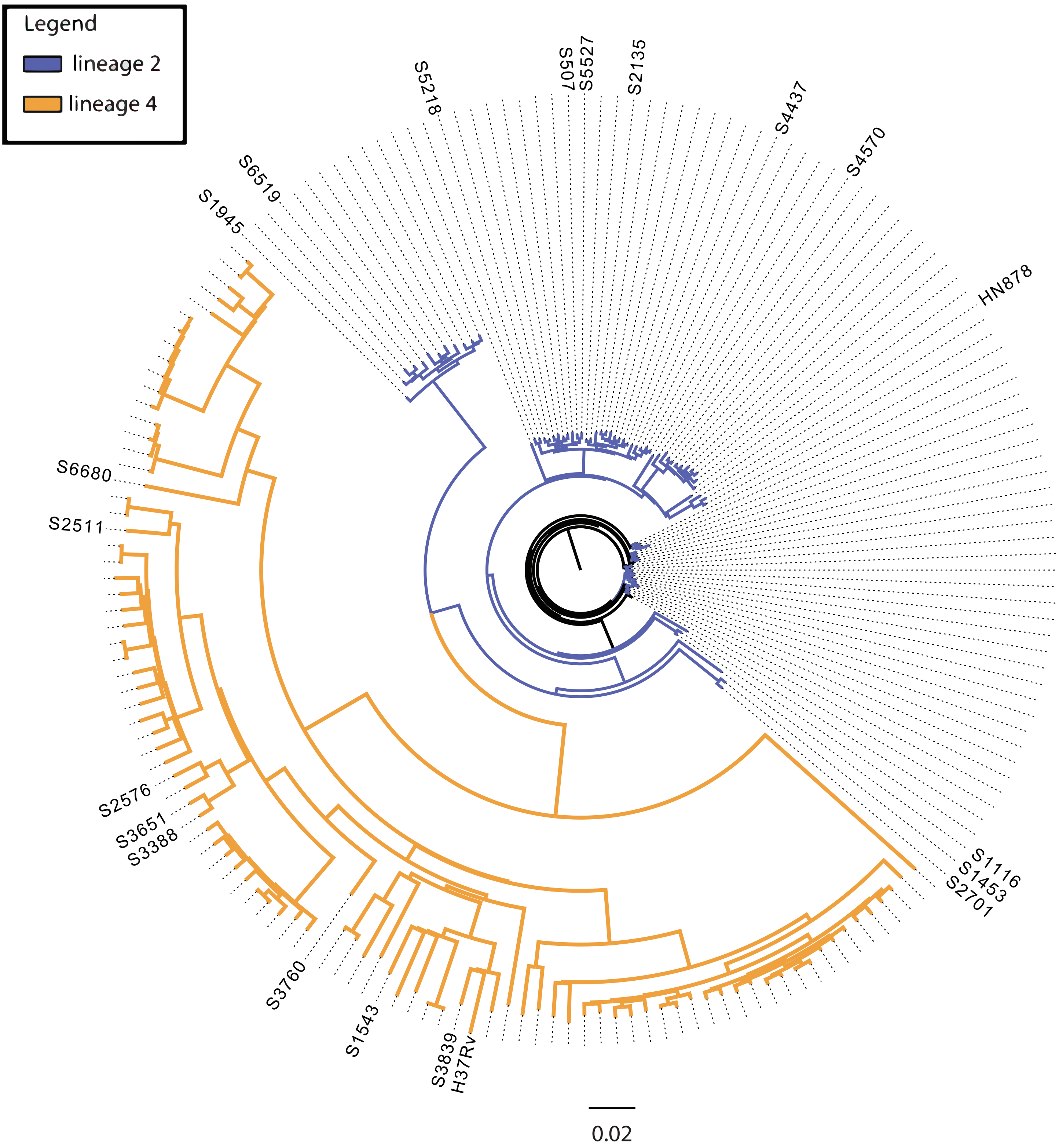

Figure S1B: Phylogenetic tree depicting all strains used in this study. This tree was constructed by aligning raw sequence reads from each clinical isolate to the *M. tuberculosis* H37Rv reference genome. This used to identify single nucleotide polymorphisms from each isolate. The construction of this tree was implemented using the same methods detailed in Figure S1A

Supplementary Figure 2

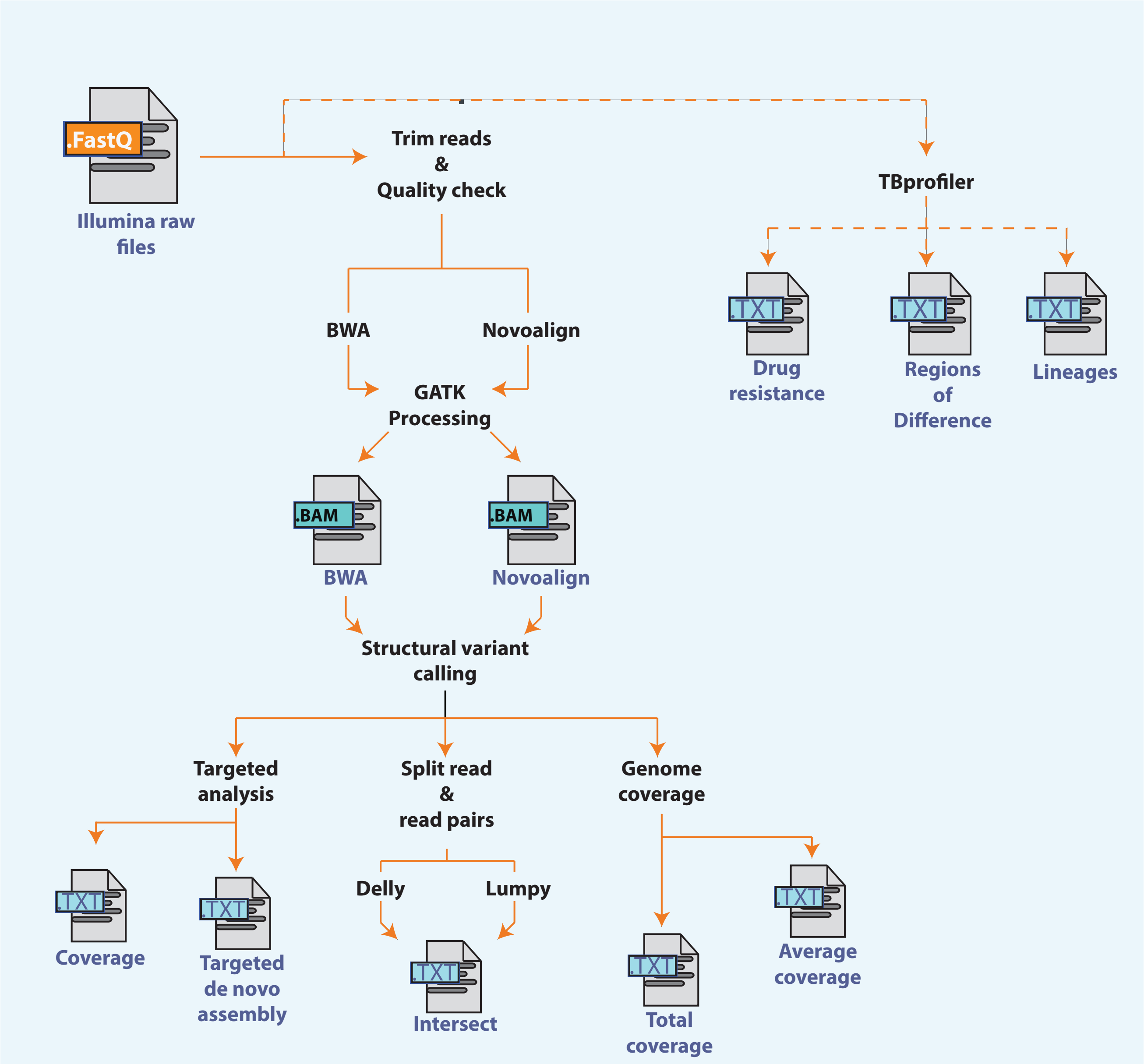

Figure S2: Schematic diagram depicting the pipeline used to analyse *M. tuberculosis* genomes. This software was constructed in Linux Ubuntu distribution using the Bourne again shell scripting language. Pre-processing and processing of Illumina raw files followed the Genome Analysis Toolkit best practises guidelines defined by the BROAD institute (https://software.broadinstitute.org/gatk/best-practices/workflow). Identification of deletions utilised both coverage based approaches as well as split read and read pair methods. Previously published software TBprofiler (dashed line) was used to determine *M. tuberculosis* lineages computationally and not created in this work. This software is available on GitHub (https://github.com/HostPathogenSU/Pegasus).

Supplementary Figure 3

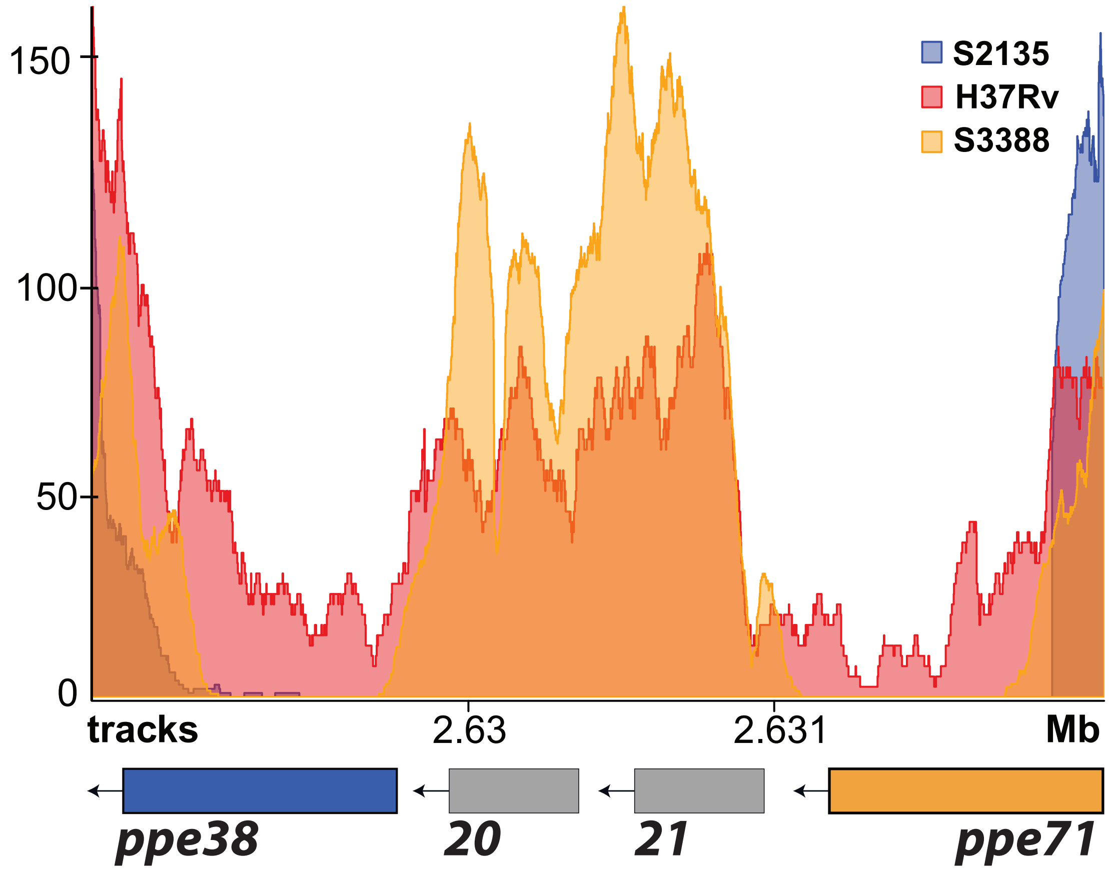

Figure S3: Reads in the *ppe38-71* region depicting three alignment profiles observed in this study. Each of the depicted strains display an alignment indicating a deletion (S2135), a wild type operon where reads fail, likely due to transposon insertion (S3388) and an example where reads are able to map across the operon (*H37Rv)*. The presence of reads in the *mt2420 (20)* and *mt2421 (21)* was considered a "wild type" operon which is able to secrete PE-PGRS proteins.

Supplemetary figure 4 A1

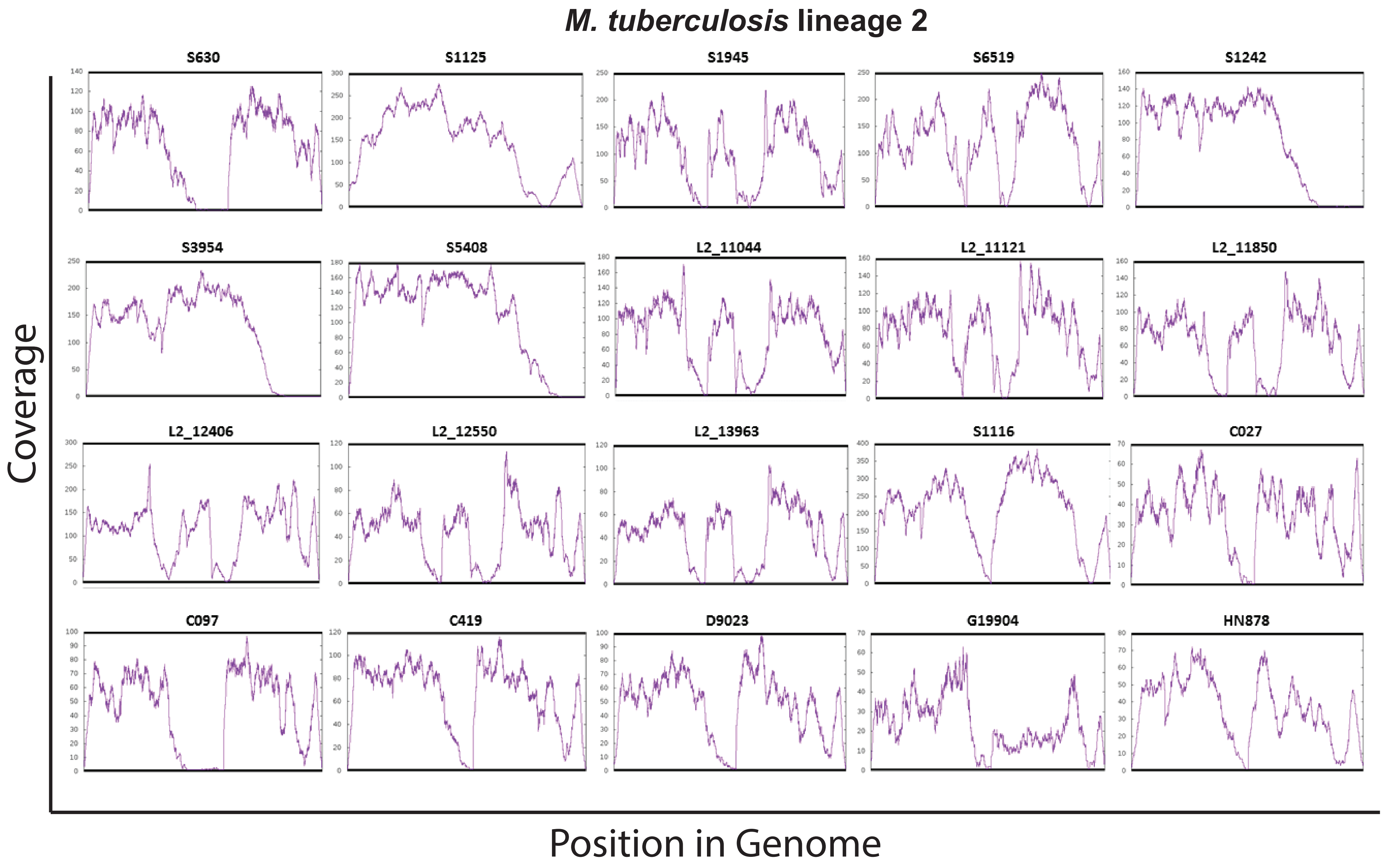

Supplemetary figure 4 A2

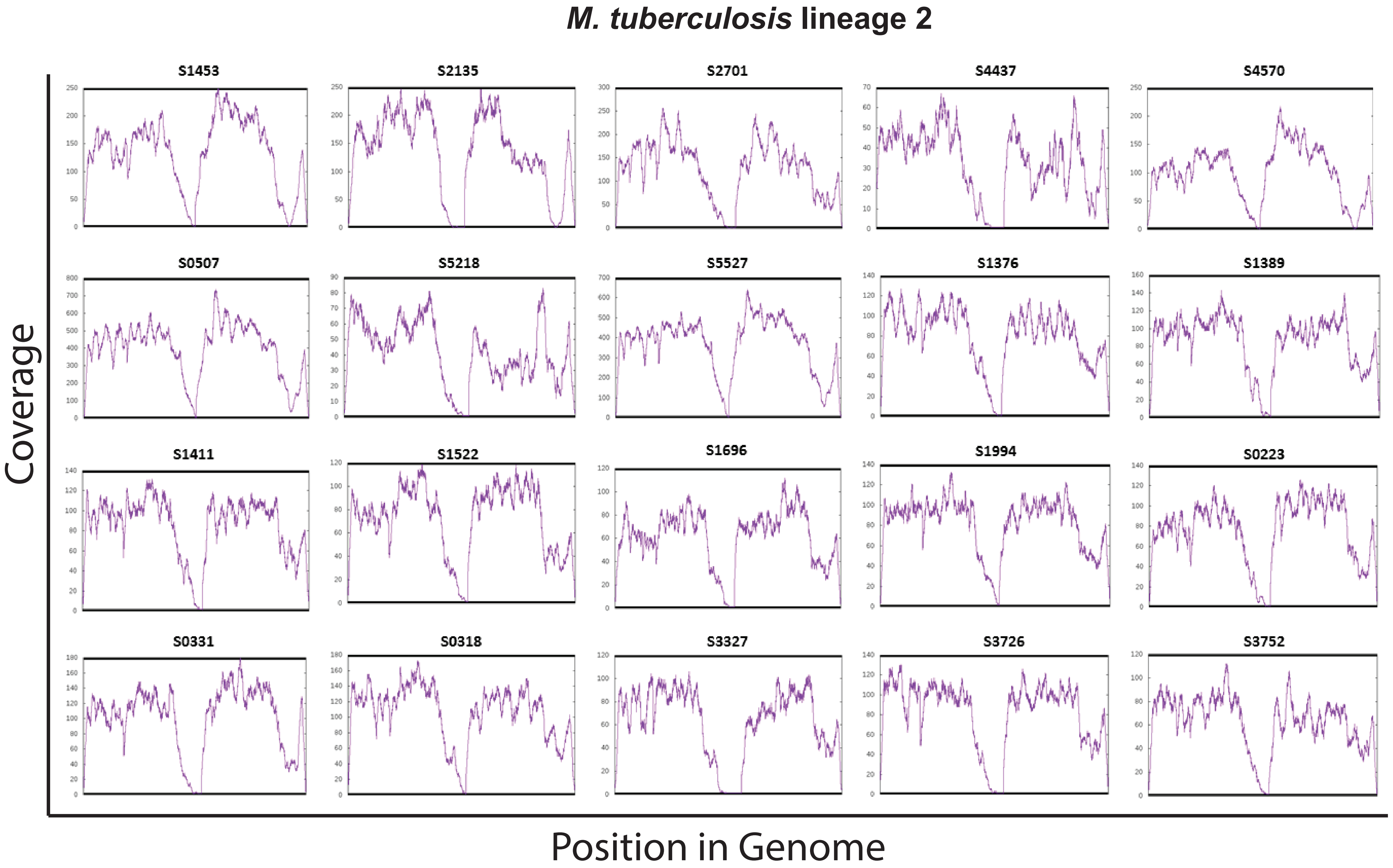

Supplemetary figure 4 A3

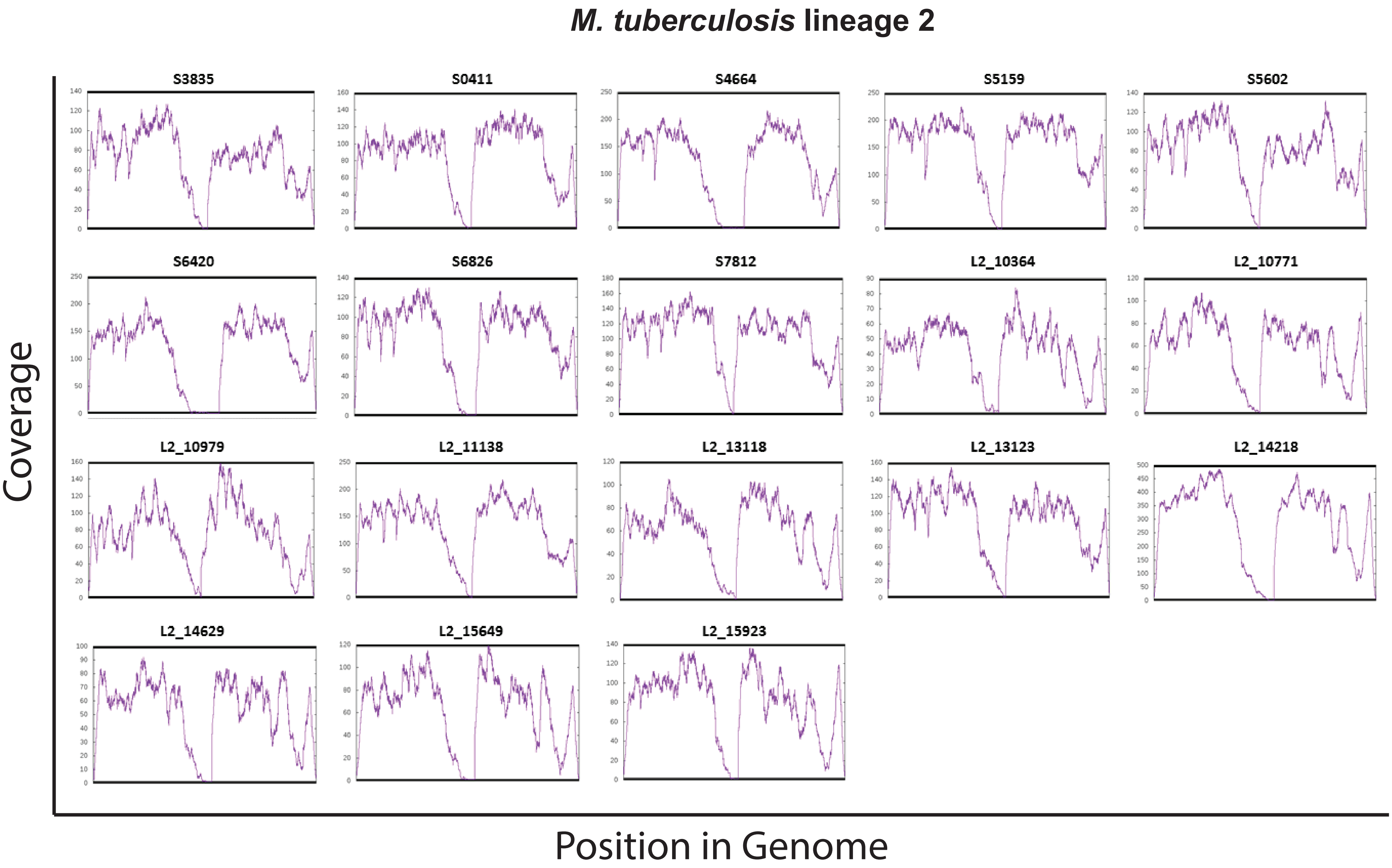

Supplemetary figure 4 A4

M. tuberculosis lineage 4

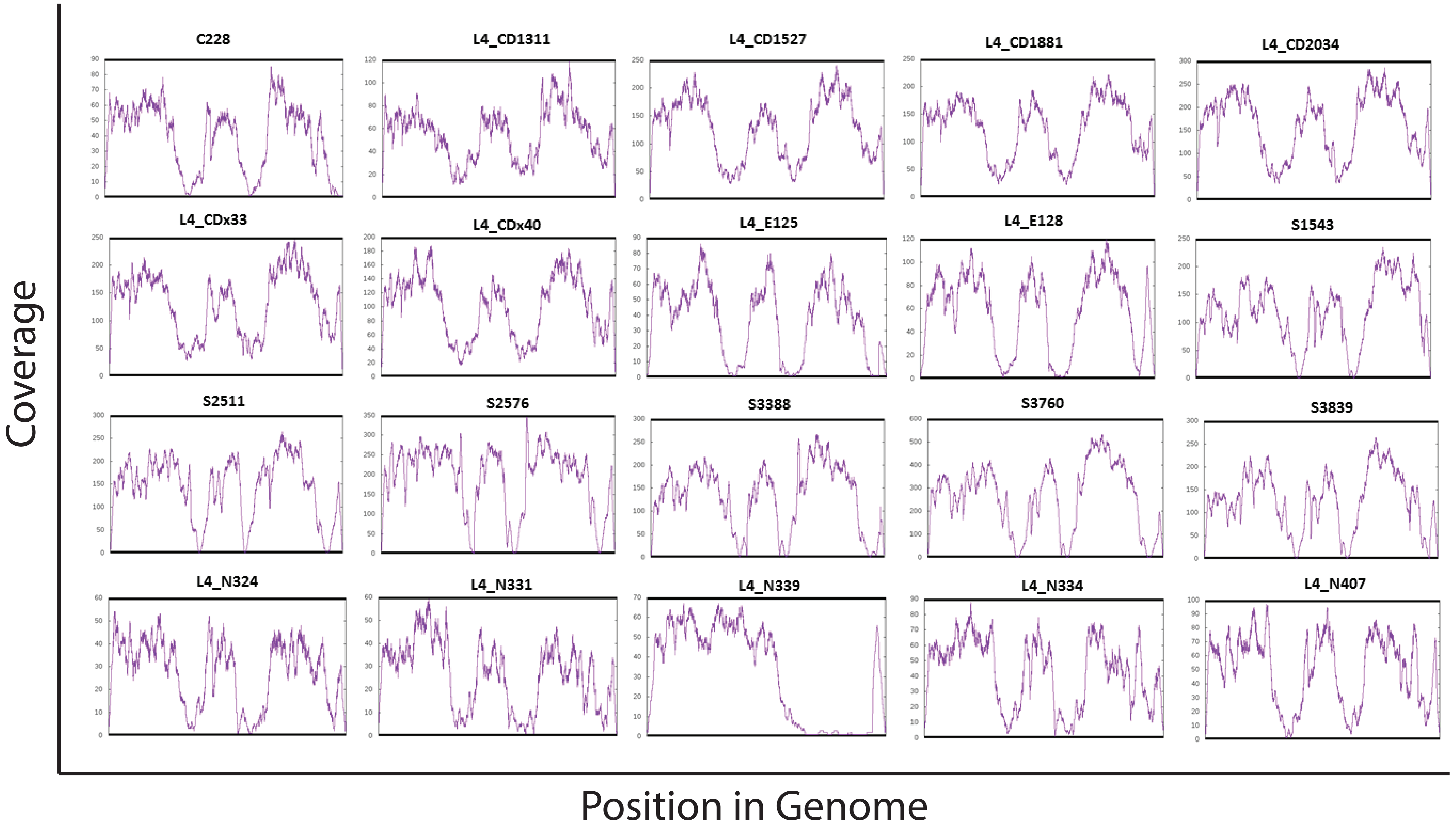

Supplemetary figure 4 A5

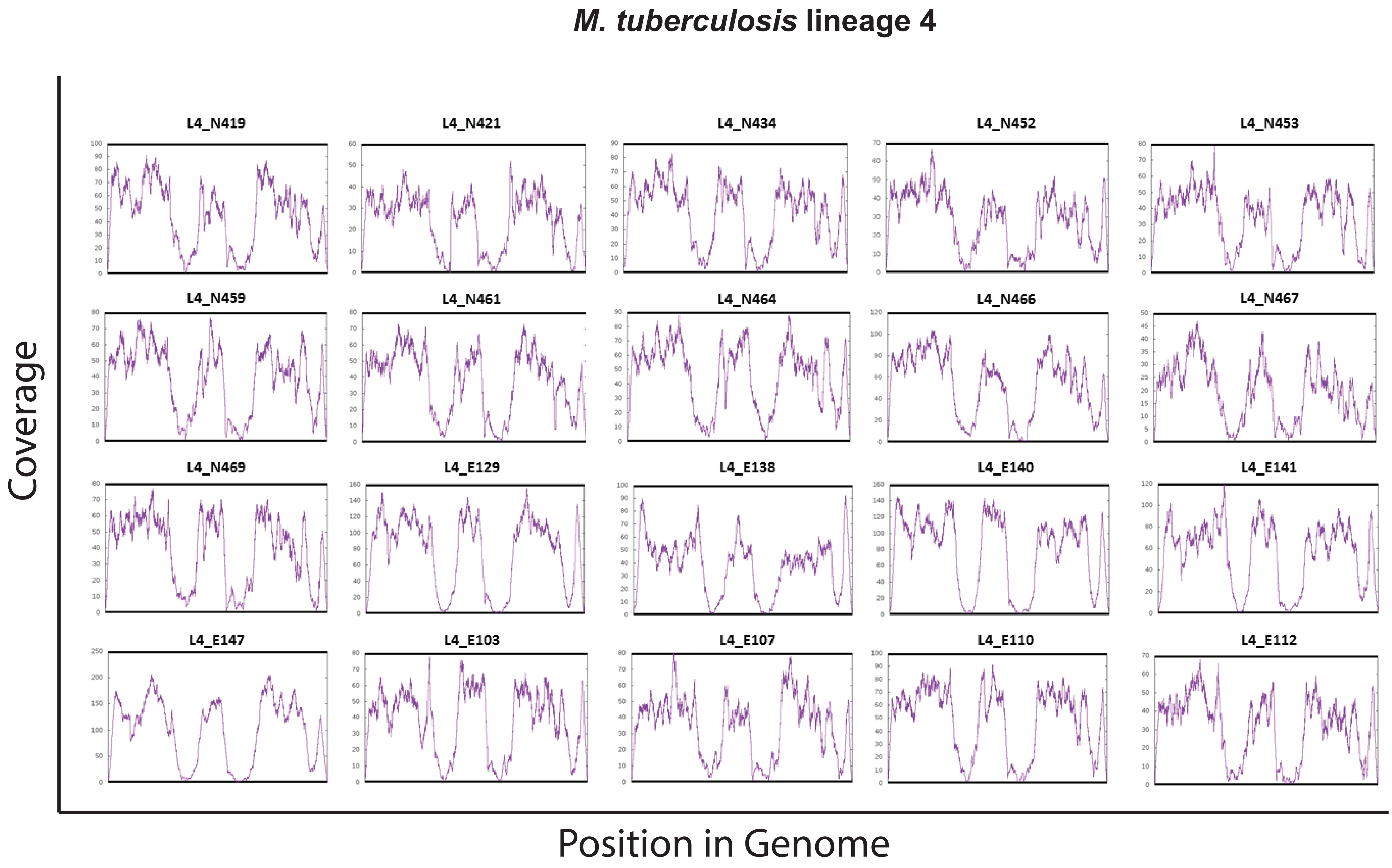

Supplemetary figure 4 A6

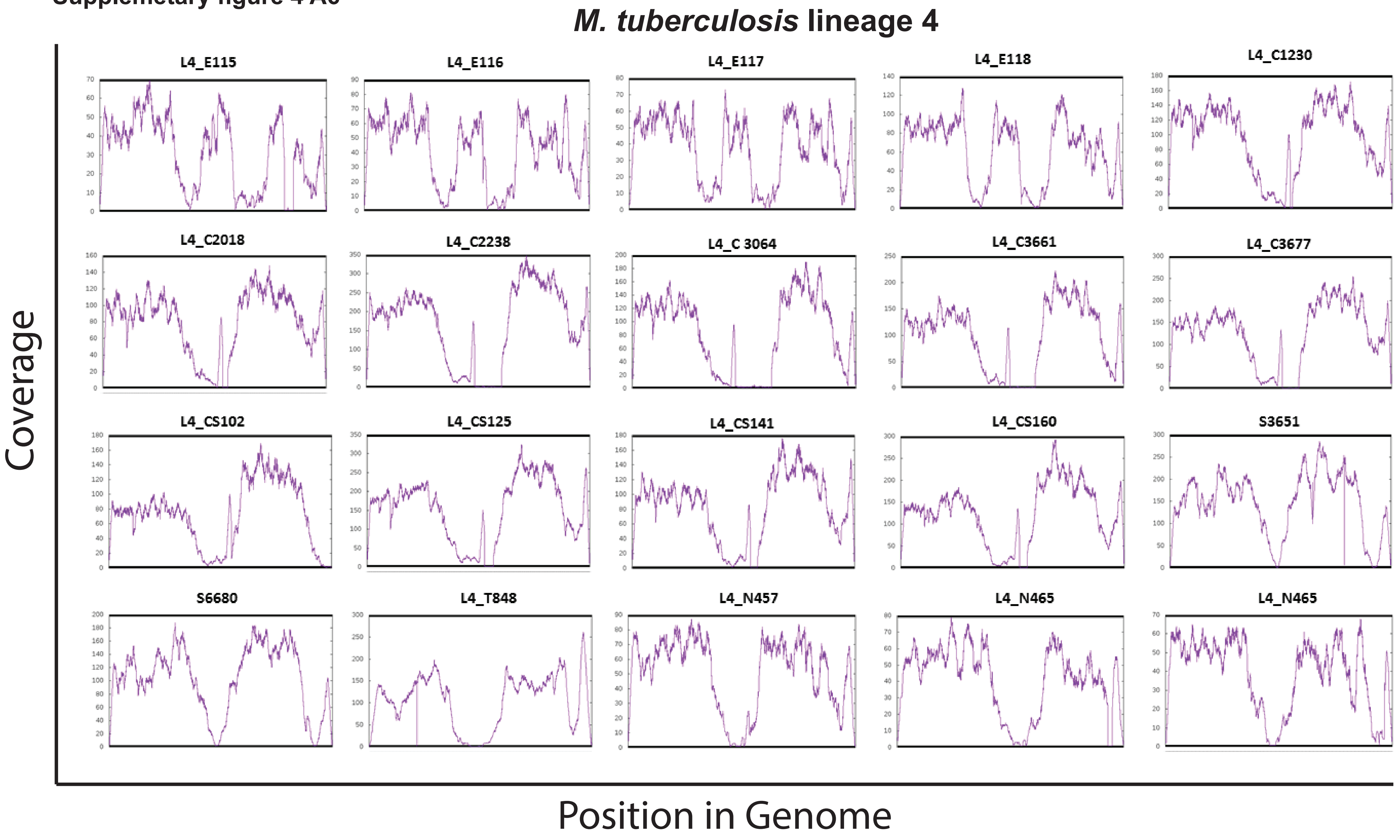

Figure 4 A1-A6: Tracks of paired end Illumina reads of 180 *M. tuberculosis* clinical isolates from lineage two and lineage four aligned to *M. tuberculosis* CDC1551. The y-axis represents the coverage and the x-axis indicates the region in the genome by base pair, starting at 2000 bp upstream of *ppe38* (position 2628768) and 2000 bp downstream of *ppe71* (position 2632079).

Supplementary Figure 5A

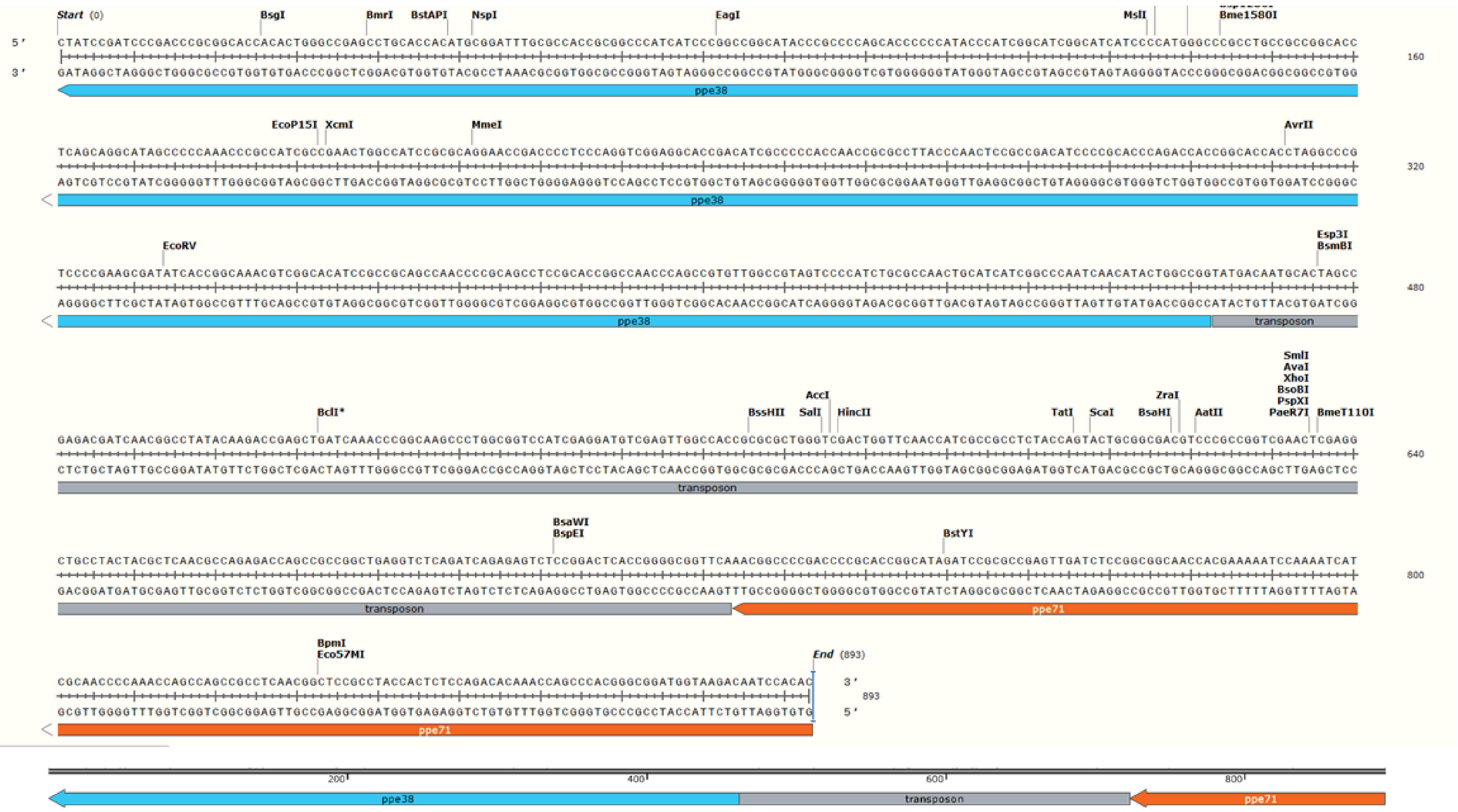

Supplementary Figure 5B

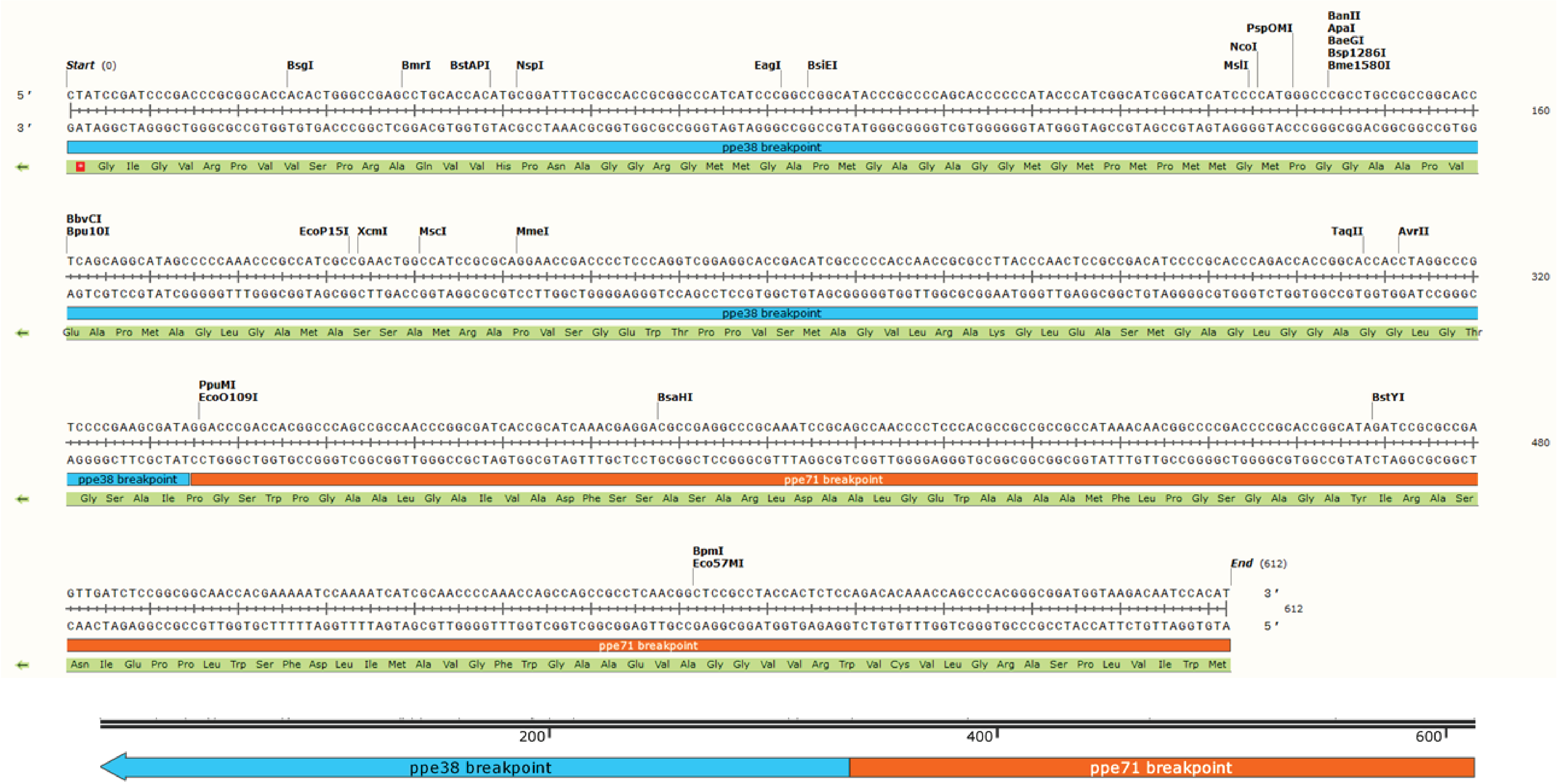

Figure S5: Schematic representation of the *de novo* assembly from S507 and S3651 in the ppe38-71 region. *De novo* assembly was done by extracting paired end reads flanking the breakpoints of the *ppe38-71* deletion on the 3' and 5' side from novoaligned BAM files from **A)** S507 and **B)** S3651 using Samtools. SOAPdenovo2 and abacas was used to perform the assembly and reconstitute the reads into a consensus sequence. Snapgene was used to visualise the assembly and annotations were based on cross comparison with the sequence known sequence information for *ppe38* and *ppe71*. The unknown sequence separating *ppe38* and *ppe71* in the S507 was identified as a transposon sequence by BLASTx.

Supplementary Figure 6

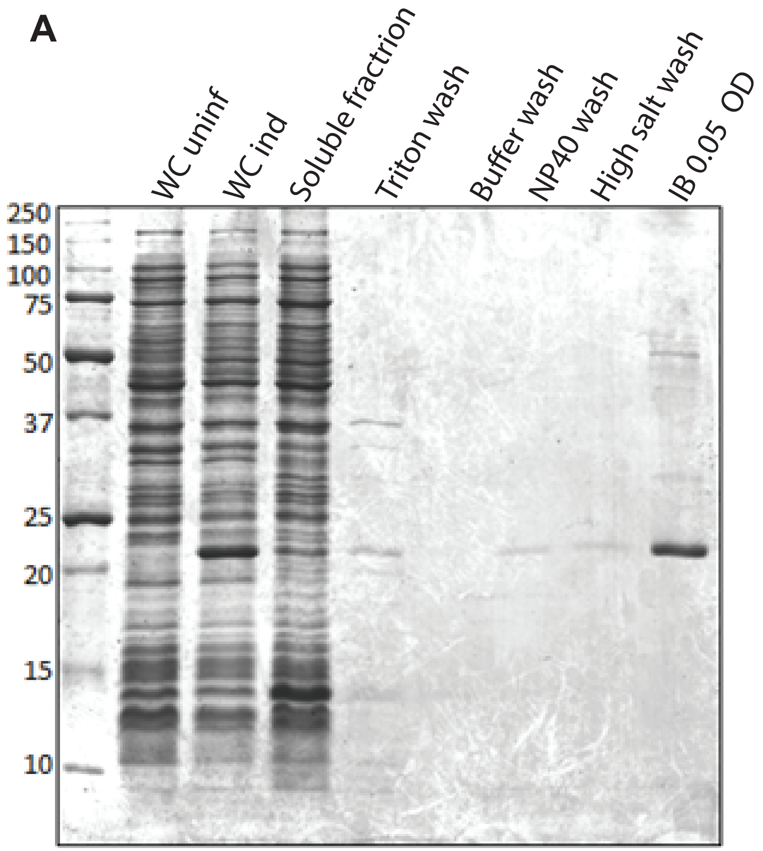

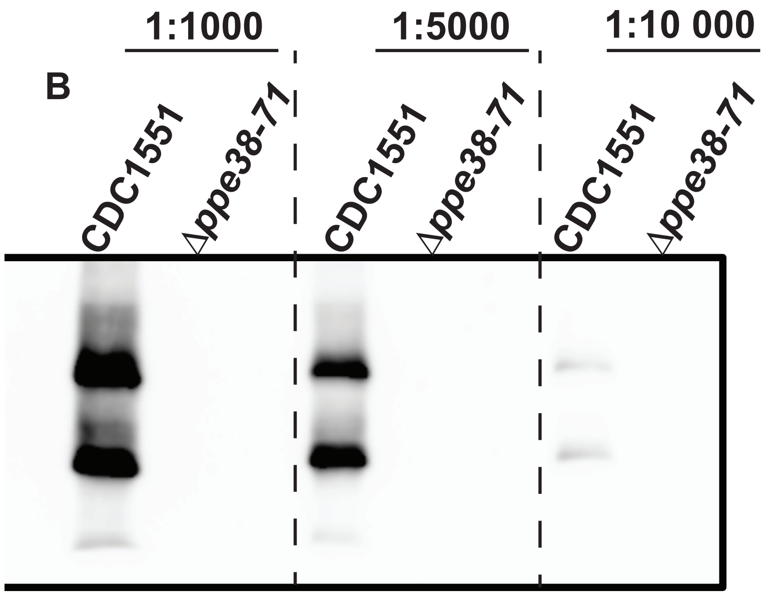

Figure S6: Isolation of ppe38 recombinant protein and testing of anti-ppe38 antiserum. A) SDS-PAGE and coomassie stain of inclusion bodies containing PPE38 protein isolated from *E. coli* top10F' cells. This inclusion body extract was used for immunisation in rabbits. **B)** Western blot depicting anti-PPE38 rabbit antiserum at various dilutions. *M. tuberculosis* CDC1551 and Δ*ppe38-71* supernatant was for as the target for western blot. Three dilutions of antibody, 1:1000, 1:5000 and 1:10 000 was used to determine the optimal concentration. Membranes were cut prior to addition of the various anti-serum dilutions, approximate positions depicted by dotted lines, and visualised at the same time for 20 second exposure. The 1:1000 dilution was used for further experimentations.

Supplementary Figure 7

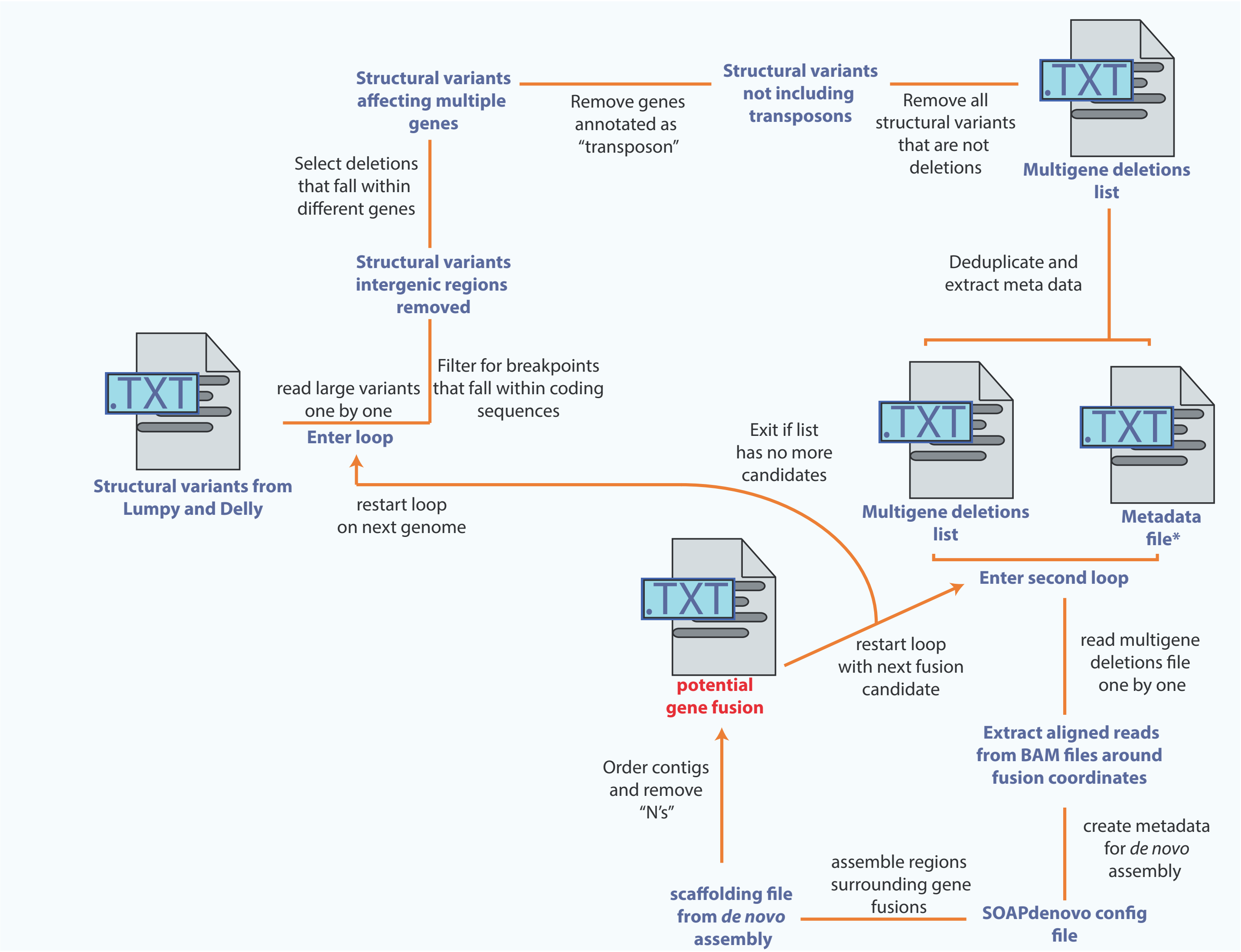

Figure S7: Schematic illustration of a sequence of events executed computationally to filter structural variant files generated by our custom illumina pipeline (figure S2). The code for this algorithm is written as an I/O operation in the bourne again shell language and implemented in the source code and can be executed from the main pipeline repository as an additional resource.

Supplementary Figure 8

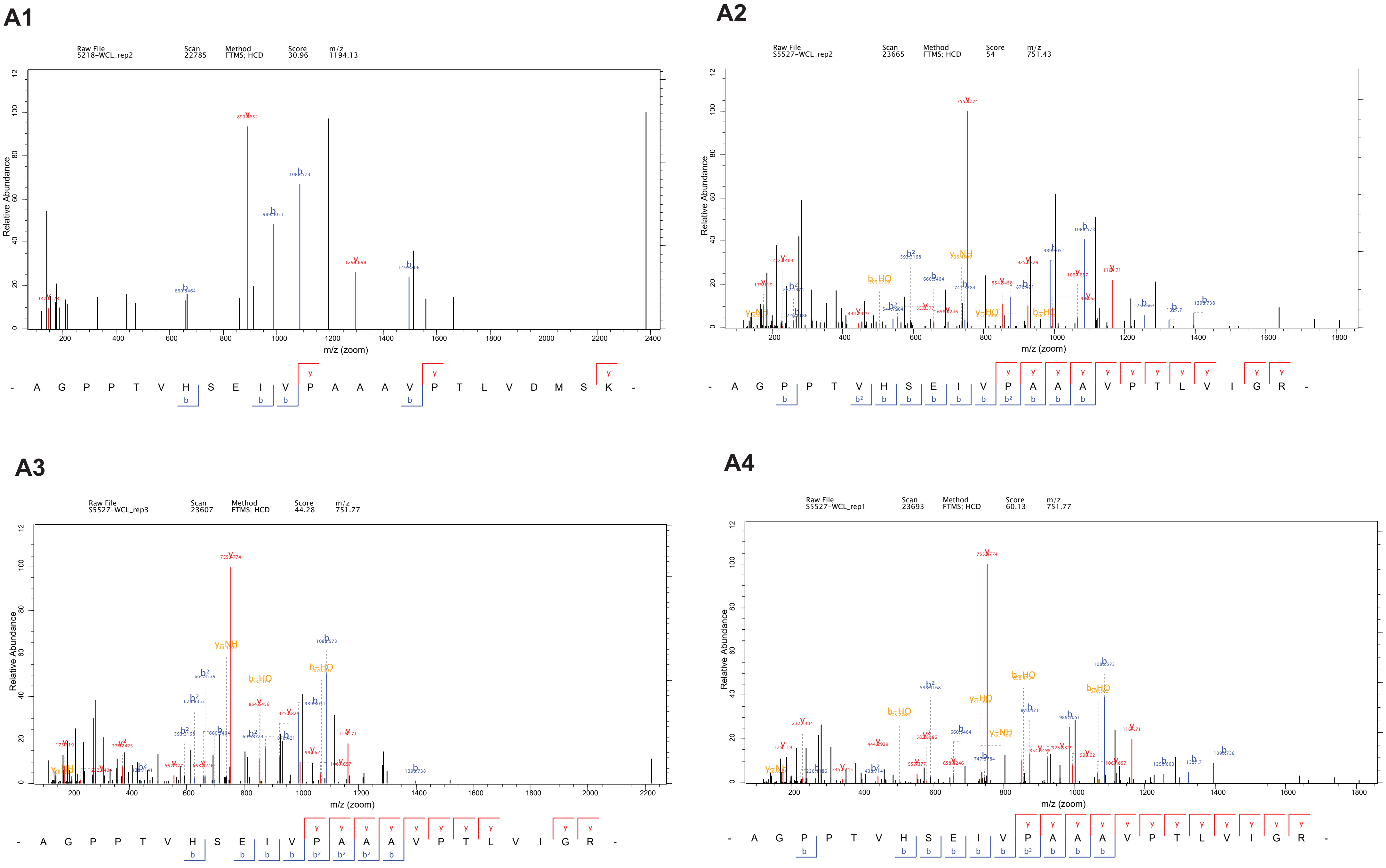

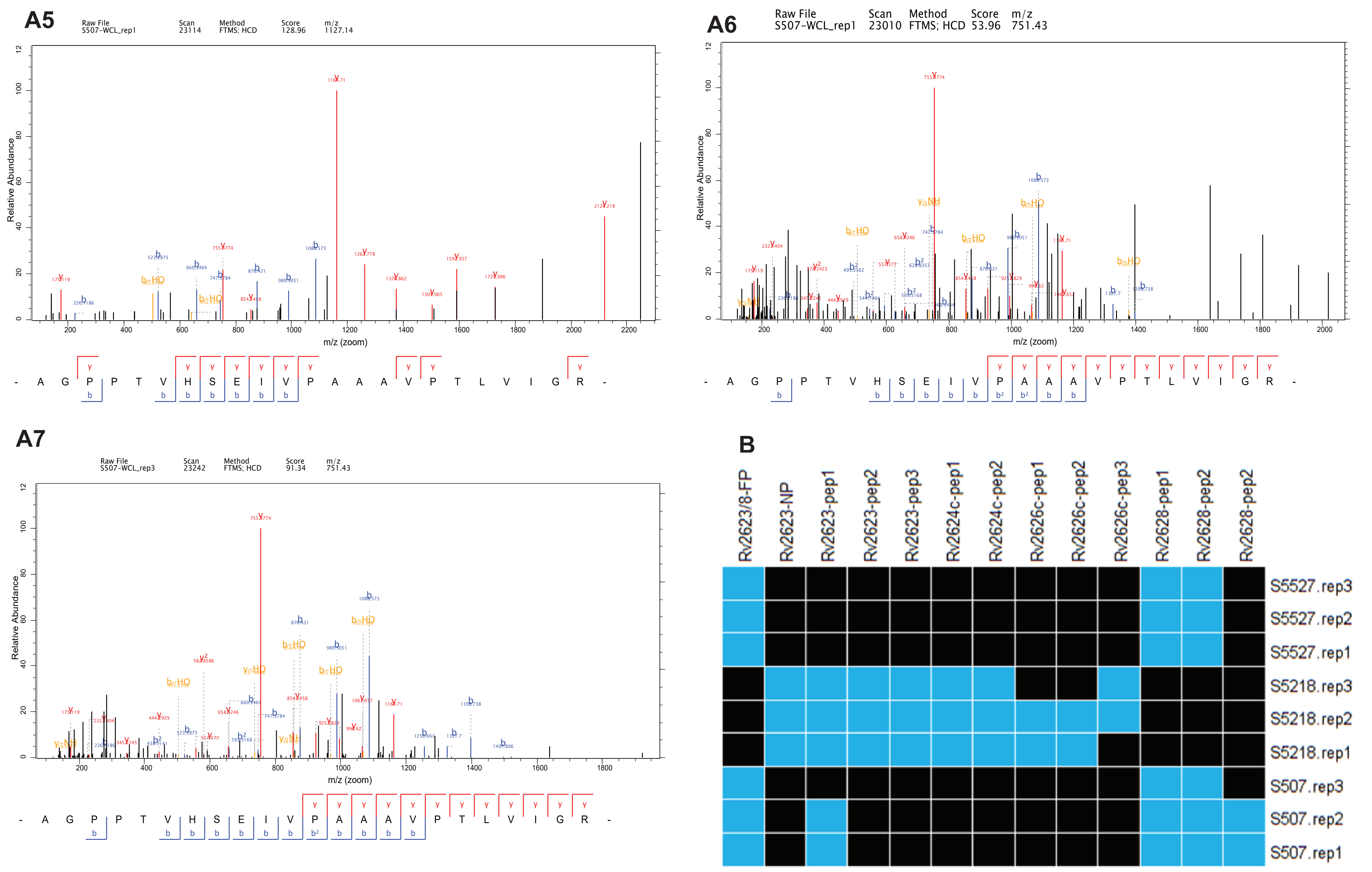

Figure S8: A) Peptide spanning the fusion junction between Rv2623 and Rv2628 with sequencing across the y1-y7 range. **B)** Heatmap depicting the identified peptides across the Rv2623-Rv2628 region. NP designates natural peptide or the wild type version in full length Rv2623 while FP designates the fusion peptide.

Supplementary Figure 9

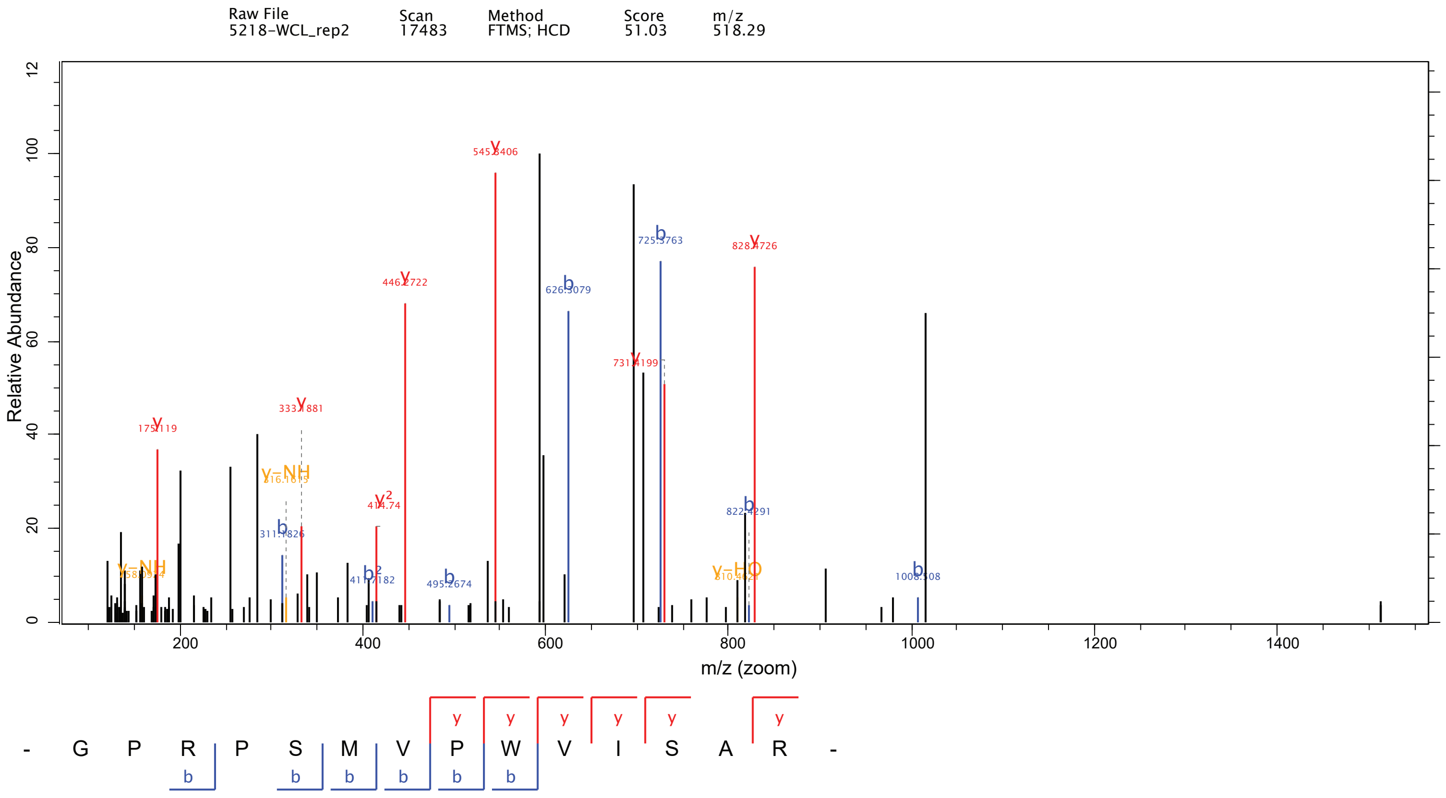

Figure S9: Tandem mass spectra assigned to a PKS15/1 fusion junction. Spectra was assigned using MaxQuant and was identified from whole cell lysates of *M. tuberculosis* S5218.
